# Quantifying *Karenia brevis* bloom severity and respiratory irritation impact along the shoreline of Southwest Florida

**DOI:** 10.1371/journal.pone.0260755

**Published:** 2022-01-05

**Authors:** Richard P. Stumpf, Yizhen Li, Barbara Kirkpatrick, R. Wayne Litaker, Katherine A. Hubbard, Robert D. Currier, Katherine Kohler Harrison, Michelle C. Tomlinson

**Affiliations:** 1 National Oceanic and Atmospheric Administration, National Ocean Service, National Centers for Coastal Ocean Science, Silver Spring, Maryland, United States of America; 2 CSS Inc. Under Contract to National Oceanic and Atmospheric Administration, National Ocean Service, National Centers for Coastal Ocean Science, Silver Spring, Maryland, United States of America; 3 Gulf of Mexico Coastal Ocean Observing System Regional Association, Department of Oceanography, Texas A&M University, College Station, Texas United States of America; 4 Fish and Wildlife Research Institute, Florida Fish and Wildlife Conservation Commission, St. Petersburg, Florida, United States of America; CSIR-National Institute of Oceanography, INDIA

## Abstract

Nearly all annual blooms of the toxic dinoflagellate *Karenia brevis* (*K*. *brevis*) pose a serious threat to coastal Southwest Florida. These blooms discolor water, kill fish and marine mammals, contaminate shellfish, cause mild to severe respiratory irritation, and discourage tourism and recreational activities, leading to significant health and economic impacts in affected communities. Despite these issues, we still lack standard measures suitable for assessing bloom severity or for evaluating the efficacy of modeling efforts simulating bloom initiation and intensity. In this study, historical cell count observations along the southwest Florida shoreline from 1953 to 2019 were used to develop monthly and annual bloom severity indices (BSI). Similarly, respiratory irritation observations routinely reported in Sarasota and Manatee Counties from 2006 to 2019 were used to construct a respiratory irritation index (RI). Both BSI and RI consider spatial extent and temporal evolution of the bloom, and can be updated routinely and used as objective criteria to aid future socioeconomic and scientific studies of *K*. *brevis*. These indices can also be used to help managers and decision makers both evaluate the risks along the coast during events and design systems to better respond to and mitigate bloom impacts. Before 1995, sampling was done largely in response to reports of discolored water, fish kills, or respiratory irritation. During this timeframe, lack of sampling during the fall, when blooms typically occur, generally coincided with periods of more frequent-than-usual offshore winds. Consequently, some blooms may have been undetected or under-sampled. As a result, the BSIs before 1995 were likely underestimated and cannot be viewed as accurately as those after 1995. Anomalies in the frequency of onshore wind can also largely account for the discrepancies between BSI and RI during the period from 2006 to 2019. These findings highlighted the importance of onshore wind anomalies when predicting respiratory irritation impacts along beaches.

## 1. Introduction

Blooms of the toxic dinoflagellate *Karenia brevis* occur along the Florida Gulf coast almost every year. This organism produces brevetoxins (PbTx), neurotoxins that accumulate in shellfish necessitating large-scale closures of shellfish beds. Consumption of shellfish containing elevated PbTx concentrations causes neurotoxic shellfish poisoning (NSP). Unlike many other marine algal toxins, brevetoxins become aerosolized when *K*. *brevis* cells break open in high turbulence (especially nearshore) and are subsequently transported onshore by winds. These aerosolized toxins cause respiratory irritation and pose a significant risk to beachgoers with chronic respiratory illnesses such as asthma [1–5], and have been measured at distances greater than 1.5 km from the shore during blooms [6]. Reports of “red tide” in conjunction with their known adverse impacts cause local residents and tourists to avoid the beaches during bloom periods. This results in significant economic losses [7–9]. Several years with particularly severe *K*. *brevis* blooms in Florida caused mass mortalities of endangered manatees [2, 10–14]. These mortalities were most acute when *K*. *brevis* blooms moved inshore during winter and spring and persisted for weeks to months. Severe bloom years were also frequently associated with bottlenose dolphin mortalities, massive fish kills, and death of sea turtles [11, 15–18].

*K*. *brevis* was identified as the cause of “red tides” off the Southwest coast of Florida in the 1940s [19, 20]. As a result of this awareness, more regular sampling of bloom events began in late 1953 (Florida’s FWC-FWRI’s HAB Monitoring Database, myfwc.com/research/redtide/monitoring/database/). Sampling was still intermittent and often event-driven until a severe event occurred in the mid-1990s (1994–1997). From 1994 onwards, blooms were sampled more systematically by the state of Florida, supplemented by various research projects [20–22]. Drawing from diverse records and sources, retrospective studies were also undertaken to fill in gaps prior to 1953 regarding the historical record of bloom occurrence [19, 20, 23–25]. These efforts utilized information such as newspaper articles, diaries, direct interviews, white papers, and historical documents. Fish kills in the Gulf of Mexico that could be attributed to *K*. *bre*vis were identified as long ago as 1528 but it was not until 1875 that fish kills were first linked to reports of human respiratory irritation [26].

Establishing a multi-year record of bloom magnitude that examines *K*. *brevis* concentrations over space (extent) and time (duration) is necessary in order to objectively evaluate the mechanisms and environmental factors most affecting initiation, growth, size, and demise of blooms. To date, the study coming closest to providing this type of record was by Feinstein [27]. She established a semi-quantitative bloom record of bloom intensity and duration from 1844 to 1955 and examined the potential role of tropical disturbances, precipitation, river discharge, and the Gulf of Mexico Loop Current on bloom formation and intensity. The study correctly identified the primary region of Florida impacted by blooms and represents a remarkable effort given the limited datasets available at the time. Many other studies have examined aspects of *K*. *brevis* bloom dynamics [28–36]. Despite the advances made in these studies, they were either of relatively short duration, or lacked quantitative measurements of either cell concentrations or spatial and temporal aspects of the blooms required to robustly evaluate the full range of factors driving *K*. *brevis* bloom phenology in this region.

Consequently, the main goal of this study was to develop and evaluate a multi-decadal *K*. *brevis* bloom severity index (BSI) to quantify the intensity of blooms through time. A bloom severity index will help assess impacts of the blooms and can be used to evaluate models of the factors causing severe, or negligible, blooms. Two existing robust data sets allowed the development of severity indices for these blooms and their respiratory irritation impacts. The first consists of *K*. *brevis* cellular abundance from the FWC-FWRI HAB monitoring database (1953–2019), and the second of respiratory irritation reports collected by the Beach Conditions Reporting System (BCRS; [37]).

The second goal of this study was to develop a respiratory irritation index (RI). Routine reporting of respiratory irritation caused by *K*. *brevis*, starting in 2006 with the development and implementation of the BCRS, has enabled quantification of this impact at coastal beach sites in Sarasota and Manatee Counties. As noted previously, aerosolized brevetoxins brought onshore by wind during bloom periods cause people to cough and experience sore throats, rhinorrhea and other respiratory irritation [2]. Coughing is the most common symptom, for even non-sensitive individuals (those without chronic respiratory conditions such as asthma). Therefore, counting coughs per unit time represents an unbiased and easily quantifiable measure of toxin exposure [37, 38].

A further argument for using the cell count and respiratory irritation data sets is the demonstrated association between the occurrence and concentration of *K*. *brevis* cells and the occurrence and intensity of respiratory irritation [7, 17, 37, 38]. *K*. *brevis* reliably produces PbTxs, so the presence of cells indicates toxin presence [39, 40]. In general, cell counts above 50,000 to 100,000 cells L^-1^ can cause slight to moderate respiratory irritation, and cell counts exceeding 1,000,000 cells L^-1^ are often associated with more severe and common irritation [38, 41].

It would be logical to assume that satellite surveillance methods currently used to monitor *K*. *brevis* blooms could constitute a third dataset to inform development of the bloom severity index. Satellite monitoring has been used routinely since 1999 [41–45]. Though effective in monitoring patterns of *K*. *brevis* blooms, the use of satellites for quantification has several concerns. The key concern is that the algorithms are not specific to *K*. *brevis*. They are based on the optical properties of phytoplankton (absorption, chlorophyll-a fluorescence, and scattering), detection of which depends on optically-deep water (too deep for light to reflect off the bottom) and low concentration of suspended sediments. Any time of the year, these algorithms can inadvertently identify non-*Karenia* blooms as *K*. *brevis*. Detection of *K*. *brevis* is more reliable in late summer and fall, when it is the most common bloom species [41]. The choice of algorithm can also influence results. Chlorophyll fluorescence can be an effective method of locating algal blooms, but high concentrations of sediment concentrations can reduce algal fluorescence in general, even while some remote sensing fluorescence algorithms can produce false positives in the presence of sediments [42]. Suspended sediment can cause backscatter-based algorithms to underestimate cell concentrations [46]. Resuspended benthic algae can also be confused with *K*. *brevis* [47]. As a result, satellite monitoring of *K*. *brevis* blooms is most successful when suspected bloom features are confirmed by field sampling. This is the approach that the National Oceanic and Atmospheric Administration (NOAA) has used in its monitoring program [41, 43, 48]. Amin et al. [49] did a time series of the estimated area of chlorophyll-a fluorescence, and assumed that this represented the area for *K*. *brevis*. However, they did not evaluate for the presence or frequency of false positives, which would be caused by fluorescence from blooms of other algal species.

Before satellite datasets are used with confidence for comparing bloom extent, intensity, and duration between years, careful evaluation of imagery for false positives/negatives of non-*Karenia* blooms throughout the seasons is needed. Another concern with using satellites is the length of record. No evaluation is possible before 1997 (OrbView-2 satellite), the start of the era of routine ocean color satellites, and chlorophyll fluorescence can only be used after 1999 (launch of Terra satellite). One area where satellite surveillance of blooms has proven useful is in assessing the long-term presence of freshwater cyanobacterial blooms, due to their distinctive spectral patterns and ability to produce surface scums (e.g. Baltic, [50, 51]; Lake Erie [52–56]; Lake Taihu [57, 58]; and South African Lakes [59, 60]). For marine harmful algal blooms (HABs), successful severity indices are rarer, but several indices have been developed based on long-term shellfish toxicity data [61–67].

The role wind plays in modulating respiratory irritation relative to bloom severity was also a focus of this study. Because respiratory distress is higher when onshore winds prevail [38] it was logical to examine the degree to which severity is modulated in periods where prevailing onshore winds are above or below the long-term average. More onshore winds favor both respiratory impacts and the occurrence of dead fish on the beach, more offshore winds favor more pleasant beach conditions. During some decades (1960s to early 1990s), sampling was largely event-driven in response to reports of water discoloration, respiratory impacts, or dead fish or other wildlife impacts. Therefore, we explored the hypothesis that wind patterns may have caused a bias in the frequency of data collection during these years. This potential under-sampling problem is important to consider when attempting to compare severity of blooms before and after 1994.

## 2. Materials and methods

### 2.1. *Karenia brevis* cell count time series

**Fig 1:**
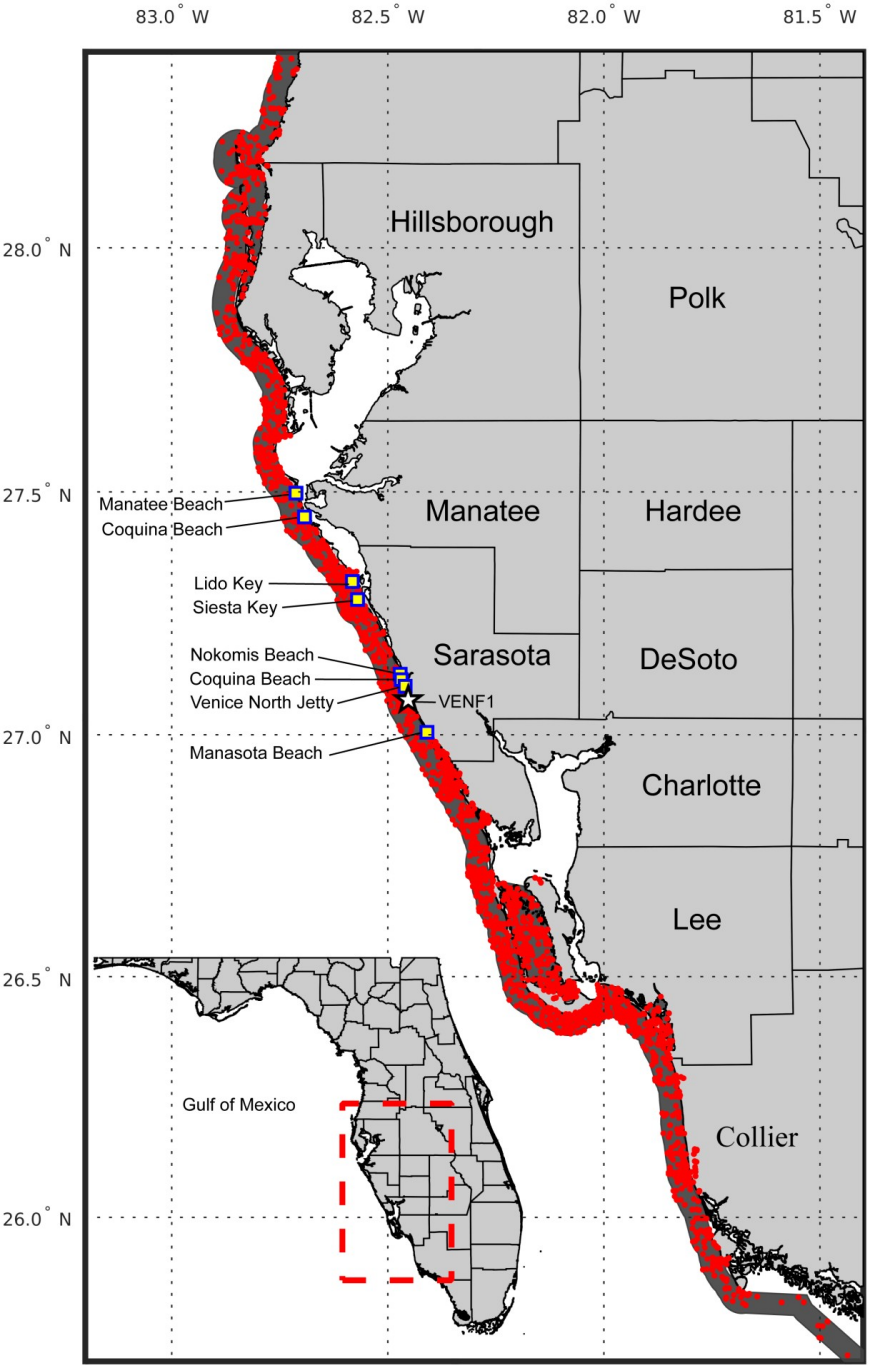
Map of the study area. Southwest Florida (25.4°-28.4°N, 82.5°-83°N), is shown in relation to the rest of Florida (Inset lower left, red box). Dark Gray shaded polygon containing red marks for cell count sample locations, shows the 5 km (3 mile) extent containing *Karenia brevis* cell counts from 1953 to 2019 used in this study. The white star in Sarasota County indicates the location for the CMAN (meteorological) station VENF1 at Venice Pier. Blue-filled squares mark the eight beach sites in Manatee and Sarasota Counties where respiratory irritation impact was monitored by the BCRS (Beach Conditions Reporting System, Mote Marine Lab).

*Karenia brevis* cell counts for all of Florida were obtained from the HAB Monitoring Database of the Florida Fish and Wildlife Conservation Commission, the Fish and Wildlife Institute (FWC-FWRI) [22]. FWC-FWRI conducts a quality control, and this quality-controlled data set was obtained as Excel spreadsheets on March 30, 2020. The data set included sample date, longitude, latitude, water depth, observed *K*. *brevis* cell counts in cells L^-1^, and county where samples were taken, if relevant. Offshore sampling was highly variable in time and space, and heavily dependent on short-term research projects. To ensure a consistent data set, cell counts collected within about 5 km (3-miles) of the coast were extracted in ArcGIS. Bays and estuaries were excluded by a straight line across their entrances (Fig 1), as bays are also inconsistently sampled, and do not directly impact Gulf beaches, where respiratory irritation is most commonly reported. The Florida Gulf Coast was extracted from the full data set, and subsequent analyses were conducted on a subset of the southwest coast of Florida, between 25.4°-28.4° N (Fig 1), which has had routine monitoring and where blooms typically occur annually. The Gulf Coast north of this latitude range has infrequent blooms and only reactive sampling [5], so it was not considered in subsequent analysis. All water depths were included in the analysis, given the proximity to the shore.

**Fig 2:**
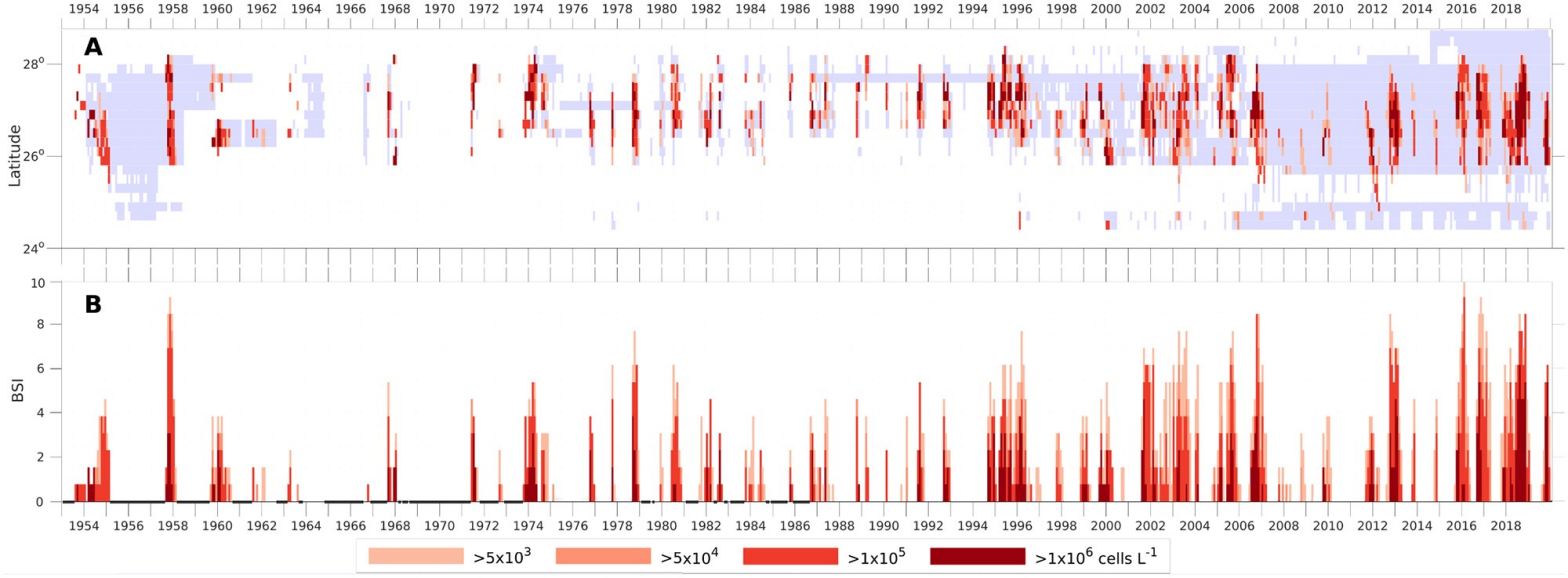
Monthly binned maxima and bloom severity index for *K*. *brevis*. (A) Hovmöller (time-latitude) diagram for the 0.2 degree latitude-binned monthly-maxima of observed *K*. *brevis* cell counts within 5 km (3 mile) offshore of the coast during 1953–2019. (B) The bloom severity index (BSI) for southwest Florida [25.4°-28.4° N] for each month determined by adding the bins for each class from the data in (A) and normalizing (more details in Section 2.1). For each bin and stack, the maximal cell count was determined and classified into five categories: <5,000 (light blue gray), >5,000 >50,000 >100,000 and >1,000,000 cells L^-1^ (salmon color to dark red). In panel (A) white indicates “no sample”, in panel (B) thicker black on the X-axis indicate months without cell count observations in the region; these occurred at intervals between 1953 and 1986. The greater the contribution of the darker colored segments to the overall height of the combined scaled values in any given month, the greater the extent of maximal cell count present.

The FWRI data set started in August 1953 and consisted of cell abundance to 2019 with concentrated sampling between Collier and Pinellas counties (geographically between 25.4°-28.4° N; Fig 1). In the 1950s, sampling was supported by several major research efforts and was relatively continuous. From the 1960s to 1993, the sampling was rather sporadic with many months not sampled at all (S1 Fig). During that time, water samples for determining *K*. *brevis* cell counts were mainly collected in response to a specific event such as a large fish kill in coastal waters, seawater discoloration, or respiratory irritation of beachgoers or boaters [20]. In 1986, Mote Marine Laboratory began routine weekly sampling in New Pass, Sarasota. For the rest of the region, routine weekly sampling along the coast began in 1995 (see Fig 2A), following directly from the monitoring of the long-lasting bloom that started in 1994.

It should be noted that samples in the last century did not discriminate *Karenia* species. The recognition of *Karenia papilionacea* [68], allowed more precise information regarding bloom composition. *K*. *papilionacea*, though toxic, appears to produce less toxin per cell than *K*. *brevis* and does not typically dominate a bloom. Since its recognition, *K*. *papilionacea* has been counted as a separate species. Consequently, counts obtained prior to 2005 may represent a slight overestimate of actual *K*. *brevis* cell concentrations.

Once the base-line cell counts within 5 km of the coastline were established, they were next binned by 0.2 latitudinal degrees, which covers about 25 km along the mostly north-south oriented coast. Finer resolution would have led to increasingly gappy data prior to 2005 (e.g., Fig 2, S2 and S3 Figs), and coarser resolution would have homogenized the cell counts in the analyses conducted on data collected after 2005. The cell count observations for each bin were further partitioned by months so that changes in cell concentrations with time of year were apparent. This combination of spatial and temporal resolutions preserved the most detail without leading to an extremely gappy dataset. *K*. *brevis* blooms are quite patchy [38, 69], and being positively phototactic, the surface concentration can change dramatically through the day. The blooms are sufficiently patchy in space and time that samples taken within 1 km of each other on the same day may have dramatically different concentrations [43, 70]. To best capture the presence of a bloom, each bin was assigned the maximal *K*. *brevis* cell concentration observed within that bin for each month. This maximal cell concentration was then assigned to one of the five categories—absent to <5,000 cells L^-1^, >5,000 to 50,000 cells L^-1^, >50,000 to 100,000 cells L^-1^, >100,000 to 1,000,000 cells L^-1^ and >1,000,000 cells L^-1^. These categories were specifically chosen to correspond to guidelines used by the state of Florida, which captures the non-linear relationship between cell counts and health impacts [41, 71]. Shellfish harvesting is prohibited when cell counts exceed 5,000 cells L^-1^, and that threshold was used here to identify bloom presence. The first two categories generally represent conditions with no to little risk of respiratory irritation. At greater than 50,000 cells L^-1^, some people may begin to experience respiratory symptoms. Concentrations from >100,000–1,000,000 cells L^-1^ may produce moderate respiratory risk in most people, and those >1,000,000 cells L^-1^ can produce severe respiratory irritation in nearly all people [38]. Consequently, partitioning the data in this manner serves as an effective proxy for respiratory irritation as well as a direct proxy for *K*. *brevis* spatial distribution (extent of the bloom) along the coast during each month over the course of the time series. The latitudinally binned monthly data set is available as S1 Data.

As some sampling gaps (empty bins) still occurred, particularly before 1994, the impacts were mitigated by using limited infilling of gaps (S2 and S3 Figs). If a bin did not contain a sample, but the bins immediately adjacent in time (month before and after) and/or space (immediately north and south) had samples, the empty bin was assigned the average concentration of the immediately adjacent bins. However, consecutive bins without samples were left empty. For subsequent analysis, this infilling helps normalize events that were sampled less than others, although, as discussed later, it does not eliminate all differences between pre- and post-1994 data.

The categorized monthly cell count data for each 0.2° latitudinal bin from 1953–2019 were next plotted against latitude from 25.4º to 28.4º N in the form of Hovmöller diagrams (see Fig 2A). The assigned categories for each monthly bin (<5,000 cells L^-1^, >5,000 cells L^-1^, > 50,000 cells L^-1^, >100,000 cells L^-1^, or >1,000,000 cells L^-1^) were color coded to represent the intensity of the bloom occurring in each monthly latitudinal bin. Light blue indicated 0 to 5,000 cells, and a graduation in color from pale to dark red was used for the categories ranging from >5,000 cells L^-1^ to >1,000,000 cells L^-1^. Bins shown in white indicate no samples were taken.

### 2.2. Monthly *K*. *brevis* bloom severity index (BSI)

To quantify the spatial distribution and intensity of the blooms, a monthly bloom severity index (BSI) was developed (Fig 2B). The index was constructed using the binned and categorized data described above. If maximal cell counts within a monthly latitudinal bin were <5,000 cells L^-1^, the bin was assigned a value of zero. The number of latitudinal bins falling into each of the four remaining categories for a given month were then tallied. These tallies were totaled for each month. Months without any field observations were indicated by black Xs on the X-axis. Next, the month with the maximal number of 0.2° latitudinal bins exceeding 5,000 cells L^-1^ was identified. This occurred in February 2016, when 13 latitudinal bins exceeded the 5,000 cells L^-1^ threshold and represents the greatest spatial extent of any bloom observed in a given month. Dividing the monthly tallies for all latitudinal bins where cells exceeded 5,000 cells L^-1^ by 13 bins (monthly maximum in February 2016), and multiplying by 10 produced the normalized bloom severity index (BSI) with a range of 0–10 over the study period (Fig 2B). BSI = 0 indicates no blooms observed along the shoreline for a given month. BSI = 10 (February 2016) represents the maximum extent of coastline impacted by a bloom present for any month during the time series thus far. For comparison and as an example, only 3 bins exceeded 5,000 cells L^-1^ in April 1963, resulting in a severity index of 2.3. (A summary of the normalization values for all the severity indices presented in this paper is provided in S1 Table.) This normalized index allows direct comparison of the BSI and the respiratory irritation index (described below in Section 2.5), although they have extremely different scales. This normalization procedure does not preclude a future value > 10, which would indicate a severity greater than any previously observed.

To capture bloom intensity each month, the relative proportion of latitudinal bins contributed by each cell concentration category (>5,000, >50,000, >100,000, >1,000,000 cell L^-1^) were indicated in the form of stacked plots. The relative contribution to the total BSI by each cell concentration category was indicated using a gradation of different colors ranging from pale to dark red. The greater the proportion of any bar within the BSI graph represented by a darker color, the more coastline impacted in a given month by higher concentration blooms (Fig 2B). The ‘neighbor’ infilling of months without samples (as described in Section 2.1) slightly increased monthly BSI in some cases when blooms were under-sampled, but did not significantly impact comparisons of variation between years (S2 and S3 Figs). The monthly data set of BSI is available as S2 Data.

### 2.3. Annual scaled bloom severity index (BSI)

**Fig 3:**
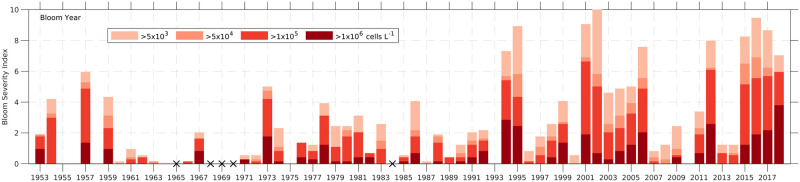
Bloom severity index for 25.4° to 28.4° N for each bloom year. The annual severity index is determined for the bloom year defined as August to the following July and uses the data accumulated from Fig 2B. The annual bloom index was normalized by the largest bloom year so it fell into the scale of 0–10 (0 indicates no bloom; 10 is the largest bloom). Black Xs on the X-axis show bloom years with no available samples.

For interannual comparisons, a normalized annual bloom severity index was computed (Fig 3). This annual BSI was determined by tallying maximal concentration categories observed in each 0.2° latitudinal bin over the course of a “bloom year”. Here, a bloom year is defined as the period from August of the specified year to July of the next. This time period generally captures the full bloom progression from initiation sometime between late summer and the end of the fall through bloom termination the following winter or spring. In extreme cases, blooms can extend into the initiation period of the next “bloom year” [32]. Because of this bloom phenology, it is more informative to partition the data by “bloom year”, rather than calendar year, as described earlier. For example, the “1953” bloom refers to the bloom during the time period from August 1953 to July of 1954. These were normalized by the maximal observed annual value (S1 Table). An annual BSI based on the calendar year is shown in S4 Fig.

The tallies of maximum concentration categories observed for all the 0.2° latitudinal bins over each bloom year were normalized by the greatest number of bins in any single year exceeding 5,000 cells L^-1^ and multiplied by 10 to produce the annual BSI (Fig 3). The maximum number of bins occurred in 2002 when 74 of the 216 possible monthly bins (18 latitudinal x 12 months) experienced cell counts exceeding 5,000 cells L^-1^. An annual BSI value of 0 indicates a bloom was not observed over the entire year in the study area and a 10 indicates the year with highest frequency of latitudinal bins with cell counts exceeding 5,000 cell L^-1^. As before, the relative contribution of bins each year with blooms exceeding >5,000, >50,000, >100,000 or >1,000,00 cell L^-1^ were color coded (pale to dark red). The greater the proportion of each bar in the annual BSI graph shown in darker colors, the more coastline was impacted by the higher cell concentrations. The Xs on the X-axis indicate years where no samples were taken (1965, 1968, 1969, 1970, 1984).

### 2.4. Seasonal bloom evolution during 1994–2018

**Fig 4:**
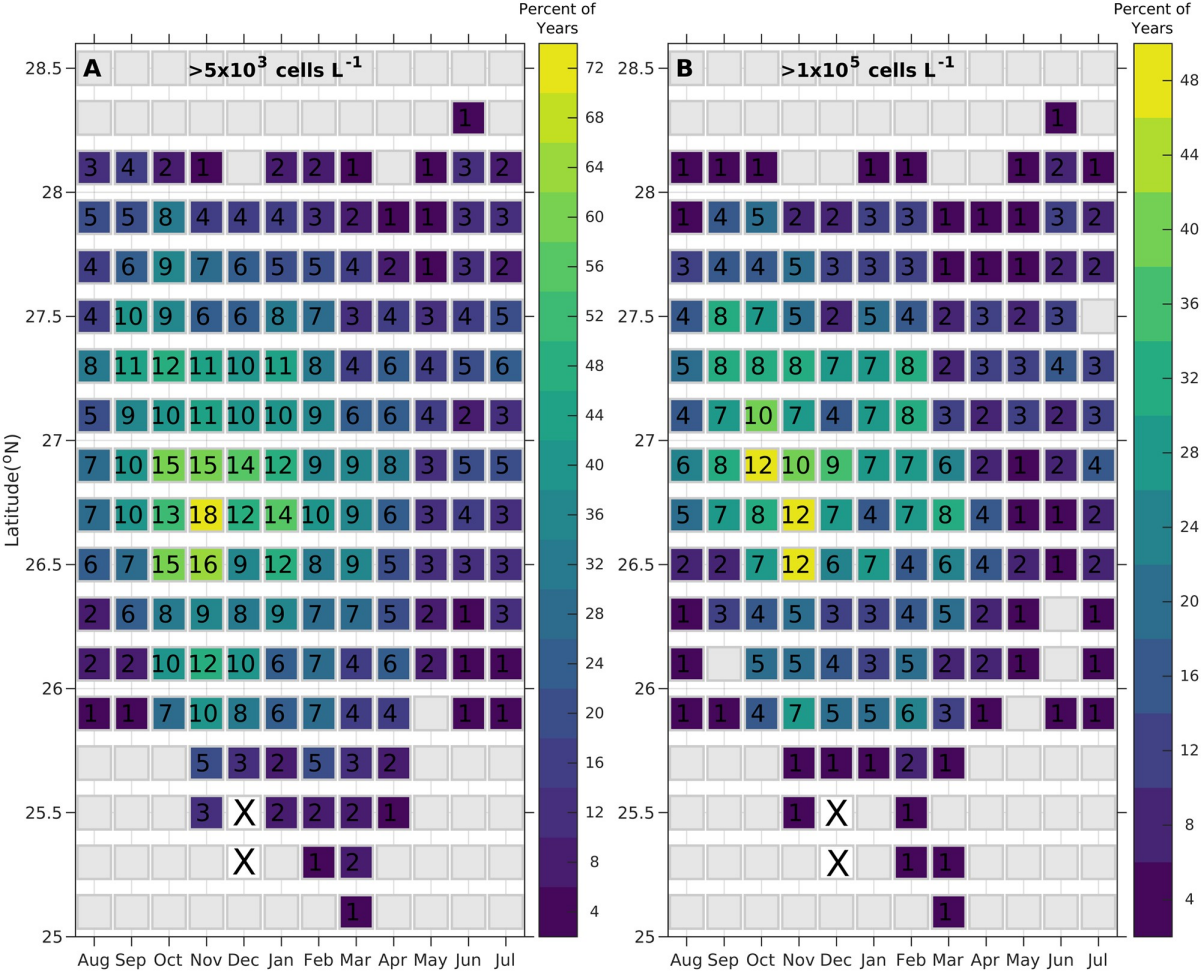
Monthly (seasonal) climatology of *K*. *brevis* bloom frequency for 1994–2018. (A) shows the frequency for > 5,000 cells L^-1^ and (B) shows >100,000 cells L^-1^. Color coding represents the percentage of years that the latitudinal bin had a concentration above the respective thresholds (5,000 or 100,000 cells L^-1^). Numbers in each bin are the number of years where the respective threshold was reached in each latitudinal bin (maximum 25). Gray indicates samples collected, but all were below the threshold. White with an X color indicates no samples were ever collected in that latitudinal bin during that month of the year. Axis runs from August to July to correspond to the bloom year. Climatology plots for thresholds of >50,000, and >1,000,000 cells L^-1^ are provided in S1 Fig.

The seasonal phenology of bloom occurrence in each 0.2° latitudinal bin was analyzed for each month from 1994–2018 to determine when cell concentrations peaked, and when they exceeded 5,000, 50,000, 100,000 or 1,000,000 cells L^-1^ in every 0.2° latitudinal bin (Fig 4). A series of four different Hovmöller diagrams were constructed, one diagram for each maximal cell concentration threshold (Fig 4 and S5 Fig). The x-axis was plotted in months to represent the bloom year (August-July), and the y-axis consisted of the 0.2° latitudinal bins. For each cell concentration diagram, the number of years during the time series exceeding the specified cell concentration was printed in each cell. Each of these cells was additionally color coded to indicate the percentage of years when that concentration threshold was exceeded.

### 2.5. Respiratory irritation risk reports collected at Manatee and Sarasota Counties

**Fig 5:**
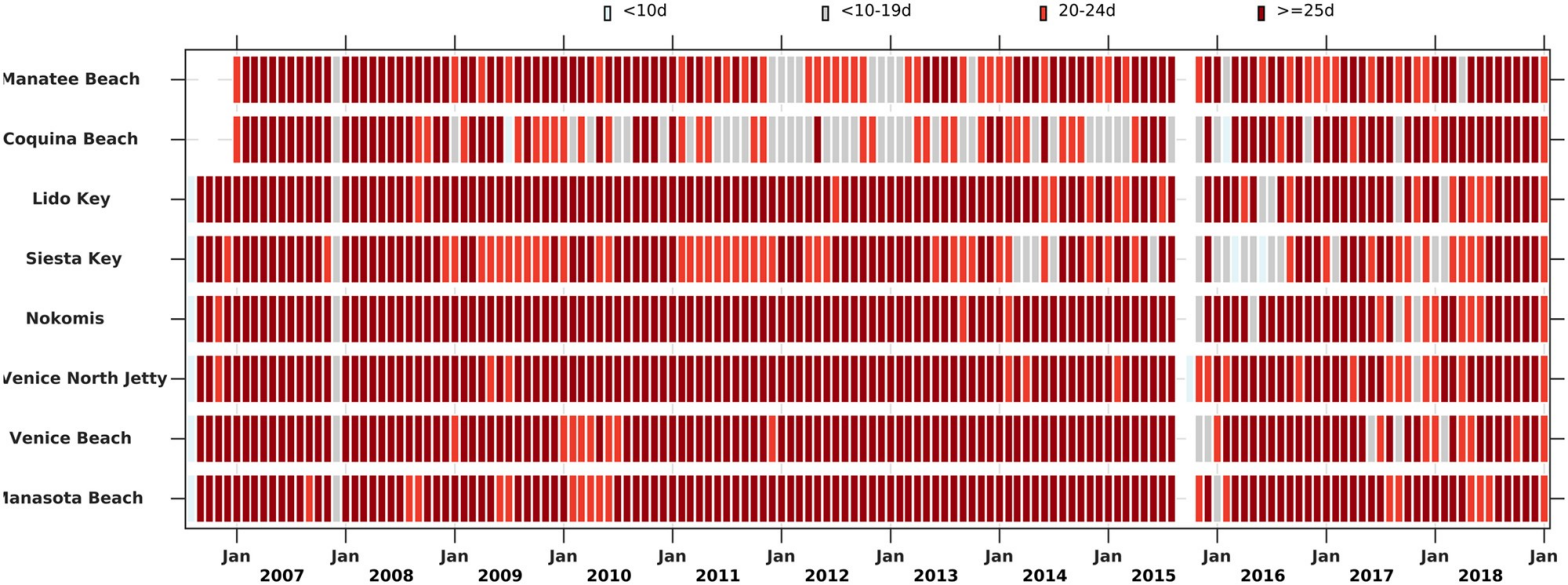
Number of days in each month with reports from the Beach Conditions Reporting System. Each month shows the frequency of days with a report on respiratory irritation condition for the eight sites in Manatee and Sarasota Counties (see Fig 1 for site locations). There was a data gap during September-October of 2015.

The Beach Conditions Reporting System (BCRS), maintained by Mote Marine Laboratory, provided respiratory irritation reports, based on the intensity of coughing, a good indicator of brevetoxin aerosol impact [37]. Respiratory irritation conditions have been routinely reported by volunteers to the BCRS database at six beach sites in Sarasota County from September 2006-January 2019, and two in Manatee County starting in January 2007 (Figs 1 and 5). These sites included Manatee Beach, Coquina Beach, Lido Beach, Siesta Key, Venice North Jetty, Nokomis, Venice Beach, and Manasota Beach (Fig 1). Reports were made once or twice daily from the same site on the beach by trained sentinels. These included report time, location, and corresponding respiratory irritation severity measure determined by the coughing frequency, namely the frequency of coughs and sneezes heard in 30 seconds [38]. The respiratory measure falls into the following four categories: none (i.e., no respiratory impact), slight, moderate, or high (severe) respiratory irritation. The number of days per month when observations were made at each of these sites during September 2006-January 2019 is shown in Fig 5. Except for Coquina Beach, most sites had 20–30 days of reports each month. It should be noted that there was a data gap during September-October of 2015 at every station. The monthly beach-day metrics are found in S3 Data.

### 2.6. Monthly respiratory irritation impact (RI) index

**Fig 6:**
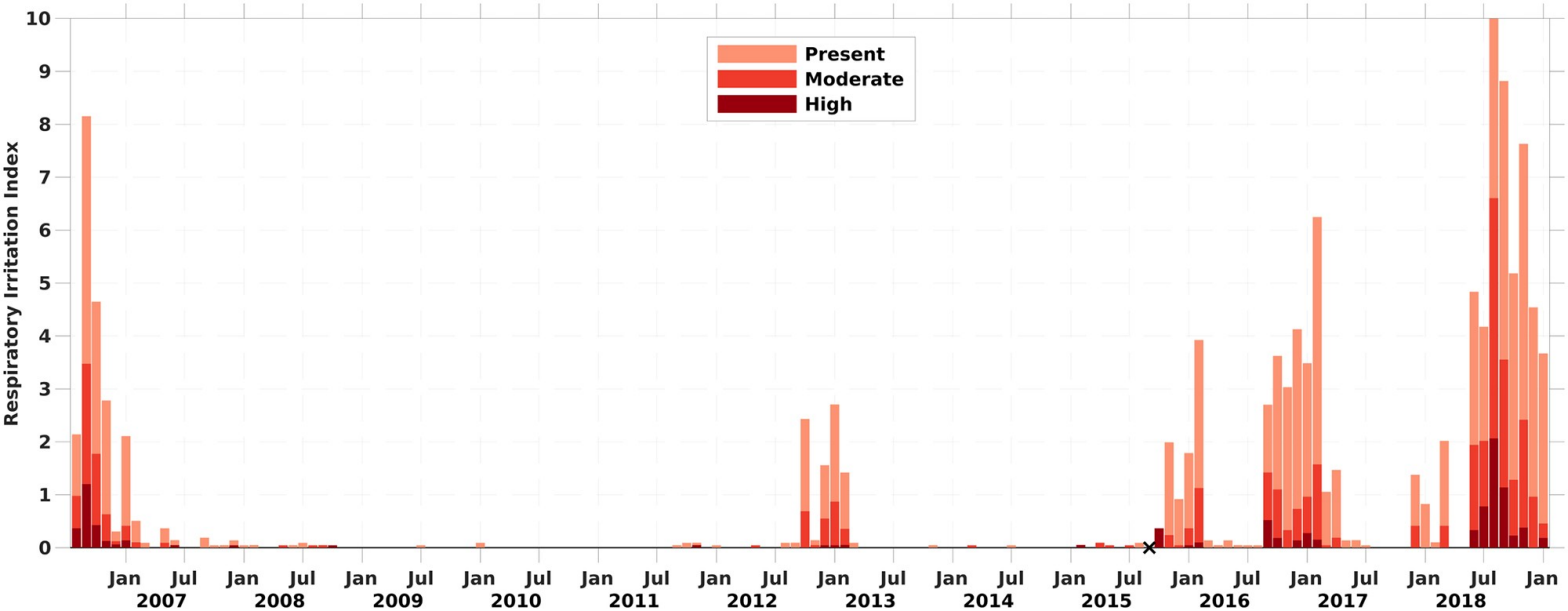
Monthly respiratory irritation impact (RI) index for Manatee and Sarasota Counties, FL from August 2006-January 2019. The RI index was defined by the number of station days for each respiratory irritation impact condition (present or slight, moderate, and high) and normalized by the highest RI to fall in a range of 0–10 (zero means that no beach experienced respiratory irritation impact, and 10 as the maximum number of beach days). Black Xs on the X-axis show months with no available BCRS data reported.

A monthly respiratory irritation impact (RI) index, shown in Fig 6, was developed using a procedure analogous to that used to calculate the monthly BSI (Section 2.2). We grouped the BCRS data into four classes of respiratory irritation: no irritation; and slight, moderate, and high irritation. For every day in a month, the number of beach sites falling into each class (e.g., irritation present) was determined. The daily totals for each class were, in turn, summed for the entire month to produce the total number of “beach-days” from all 8 sites that fell into each respiratory class over the month. These totals were then normalized by dividing by both the number of sites (8) and the number of days in a given month. This yielded the proportion of beach-days for each irritation class within each month. The beach-days per month for each respiratory irritation level was then divided by the largest monthly proportion value observed of the “irritation present” class (0.88 in August 2018, which had 218 beach-days out of a possible 248) (S1 Table). Lastly, the resulting fractional values were multiplied by 10 to produce an RI index varying from 0 to 10, where 0 indicates none of the beaches experienced respiratory irritation during the month, to 10, which was the month (August, 2018) exhibiting the greatest number of beach-days with respiratory irritation observed at any time during the study period.

The resultant monthly RI time-series for the three categories is presented in Fig 6. The height of each monthly bar indicates the relative respiratory impact at Sarasota and Manatee Counties beaches, relative to the maximal impact observed over the study period. This index is comparable to the BSI index shown in Fig 2B. The higher the total bar isfor each month, the more extensive the respiratory impact in both space and time. The fraction of each monthly RI contributed by the three RI categories was assigned the same color scheme as used for the BSI—light red for RI present (slight), red for moderate RI, and dark red for high RI. The greater the overall contribution of the red or dark red bars to the height of the monthly index bar, the more severe the relative respiratory impact of the bloom. The monthly RI data set is found as S4 Data.

### 2.7. Annual respiratory irritation impact index

**Fig 7:**
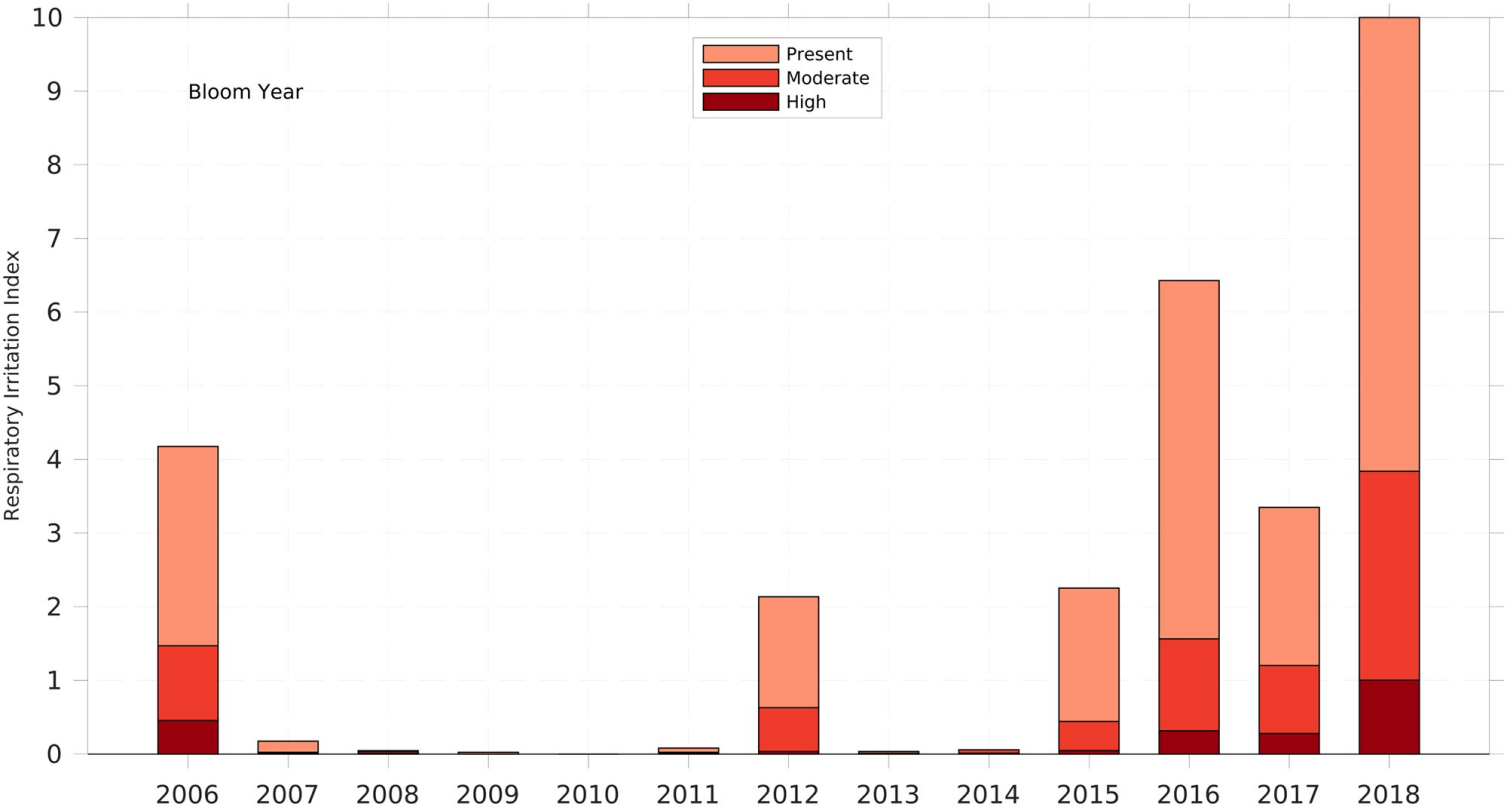
Respiratory irritation index for each bloom year for Manatee and Sarasota Counties, 2006 to 2018. The bloom year starts in August and ends the following July, e.g., 2006 includes August 2006 to July 2007. The respiratory irritation index was defined by the total beach days over the year of each irritation condition (present, moderate, and high), and scaled such that the year with the maximal frequency had a value of 10, and zero indicates no respiratory irritation at any beach during the year.

An annual respiratory irritation impact index (RI) was calculated using a method analogous to the annual BSI (Section 2.3). The beach-days of respiratory irritation classes were accumulated over the bloom year (August to next July). These summed values were normalized by the largest annual proportion of the “irritation present” class observed in any single year. The maximum proportion of beach-days (0.29) occurred in the 2018 bloom season, with 856 of 2,920 annual beach-days (365 days multiplied by 8 beaches) (S1 Table). The proportions were then multiplied by 10 to produce the annual RI index, which ranges from 0–10. The resulting RI values for each year were plotted as detailed in section 2.6 (Fig 7). As before, the light to dark red colors comprising each bar in the annual RI graph represent the relative proportion of the RI belonging to each respiratory class (absent, present [slight], moderate, and high irritation). For the interest of readers, a calendar year RI is shown in S6 Fig.

### 2.8. Beach specific, seasonal frequency of respiratory irritation

**Fig 8:**
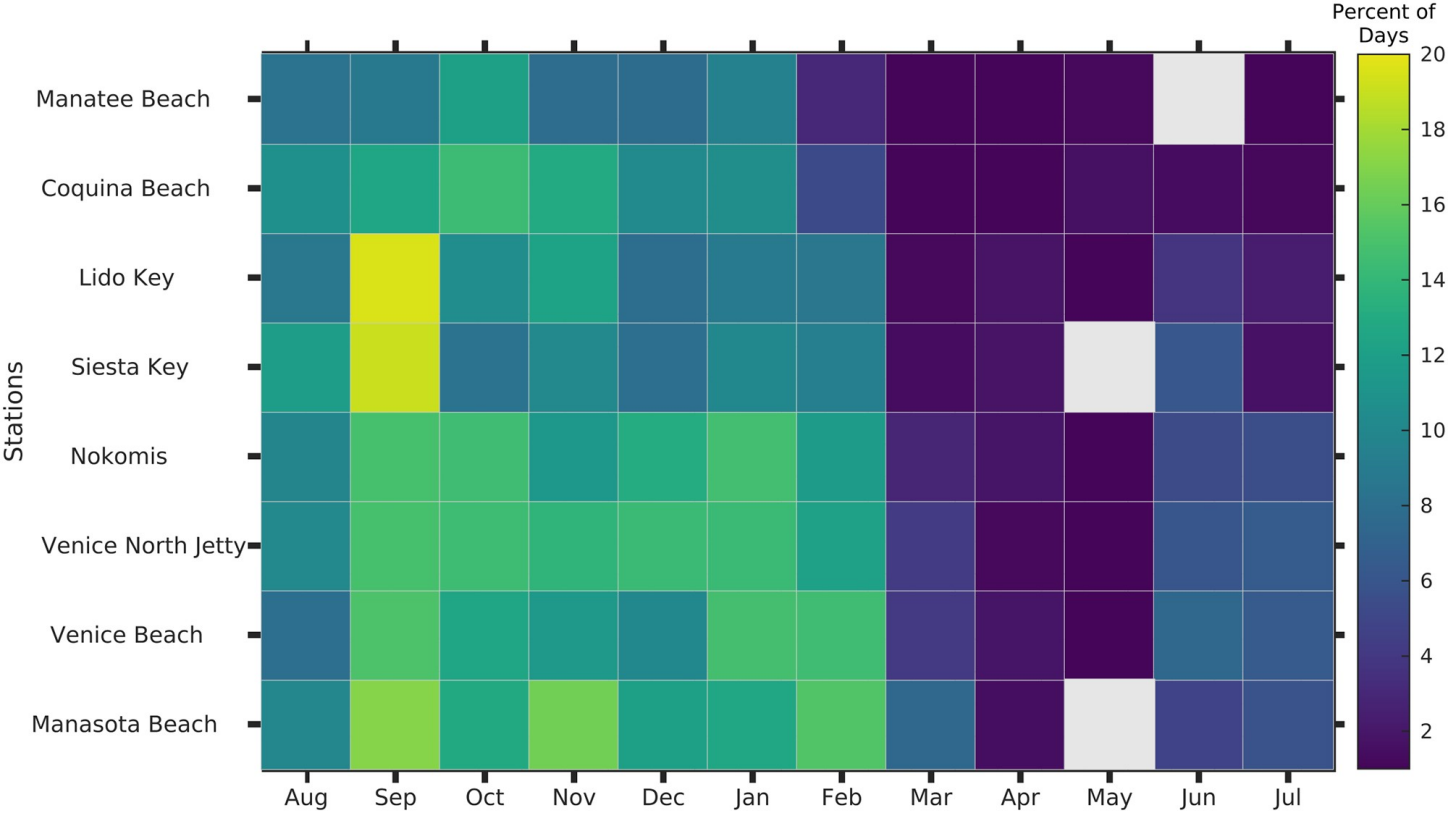
Monthly (seasonal) climatology of the frequency of respiratory irritation. The frequency is the percentage of days with respiratory irritation observed at each beach for each of the twelve months over the data set from 2006–2019. Stations are ordered from north at top to south at bottom (locations in Fig 1). Gray color indicates months when the beach did not have detectable respiratory irritation.

The seasonal evolution of respiratory irritation risk between 2006–2019 for each of the eight beach stations in Sarasota and Manatee Counties was quantified in the form of a Hovmöller diagram (Fig 8). The x-axis is month, the y-axis consists of the BCRS beach stations ordered from north at the top to south at the bottom and the cells are color coded to show the percentage of days in a month (relative to the total number of days when BCRS reports were available) when respiratory irritation impact was present. Gray color indicates months which had no reported respiratory irritation impact for that beach. This information is relevant for developing improved approaches to assessing the economic impacts of *K*. *brevis* blooms (Fig 8).

### 2.9. Analysis of the relationship between bloom severity and annual prevailing winds

Onshore winds are well-recognized as a modulating factor of respiratory risk [38, 72–74]. Onshore winds transport aerosolized toxins onto the beach during *K*. *brevis* blooms, heightening risk, while offshore winds may reduce or eliminate risk. A goal of this study was to assess how respiratory severity compared to the bloom severity, and accordingly identify the degree to which respiratory impact was affected when onshore wind frequency was anomalously high or low. This required developing a wind speed and direction time series. Two sets of relevant sources of wind records were used in the analysis. The first was observations by the NOAA Coastal Meteorological Automated Network (CMAN) Station at Venice Pier, FL (VENF1; white star in Fig 1), which contained continuous wind records from the late 1990s to the present and were representative of conditions at Sarasota County Beaches [75]. We used the standard meteorological records, which include hourly wind speed and direction. The second source is the North American Regional Reanalysis (NARR, 2020), which covers 1979 to the present. The NARR data set is a data-assimilative product created by NOAA’s National Center for Environmental Prediction (NCEP) using atmospheric model simulations over North America with a spatial resolution of ~30 km and a temporal resolution of 3 hours. NARR accurately represents the wind variation over the continental United States [76]. To validate how well NARR corresponds to the VENF1 measurements, the two data sets were compared over the period from 1990 to 2018. Because NARR is collected every 3h, the VENF1 data set was subsampled to match the NARR times. Because NARR has a 30-km grid, the NARR winds from the grid points around VENF1 were interpolated to derive the simulated wind at VENF1. Results of the comparison showed that VENF1 and NARR onshore winds agreed 81% of the time. Offshore winds agreed 89% of the time. These results showed that the NARR data set serves as a good proxy of observed wind conditions near Sarasota County.

As discussed earlier, prior to 1994, *K*. *brevis* field sampling was less frequent because it was carried out in response to reports of “red tide”. In order to examine if wind was a factor in the less frequent sampling, we examined two time periods. The first started in 1979, when NARR became available and extended until 1994 when systematic sampling started. The second covered 2006–2018 when the BCRS started. For these analyses, NARR was used for 1979–1994, while the continuous VENF1 was used for 2006–2018. These data sets can be used to assess whether periods with more frequent offshore winds correlate with the periods where blooms were not sampled as frequently. Onshore wind frequency (OWF) was calculated from these two time series using the following procedure. First, the hourly VENF1 and 3-h NARR winds were decomposed into onshore and alongshore components. The monthly time series of OWF was calculated by the total number of hours within each month when winds blew onshore, divided by the total number of hours in that month for which wind observations were available. This yielded the fraction of the time each month when winds blew onshore. OWF anomalies (OWFA) were computed by taking the OWF for each month of the time series and subtracting the long-term-average OWF for that month. Positive (or negative) OWFA indicated that the onshore winds were more (or less) frequent than average for that month. The OWFA monthly data sets for NARR and for VENF1 are available as S5 and S6 Data.

## 3. Results

### 3.1. Bloom intensity and duration

For the first time, both the temporal and the spatial distribution of *K*. *brevis* blooms and sampling frequency along south Florida are presented (Figs 1 and 2A). The change from reactive to systematic sampling in 1994 is apparent. Despite sampling gaps prior to 1994, the time series confirmed that blooms occurred in most years, as previously described [20, 77]. The longest documented blooms occurred in 1994–1996, 2001–2003 and 2017–2019, all of which occurred after more routine sampling started. The monthly and annual BSIs show that most of the large blooms occurred after 1994 (Figs 2B and 3). While this difference could be attributed to an actual increase in bloom size and duration, it may also be explained by increases in the spatial coverage and temporal frequency of sampling (Fig 2). Large blooms were noted in 1957, which was robustly sampled, and in 1973 and 1978, which were not robustly sampled. Blooms with the highest recorded cell densities took place in October 1957, October 2006, and in February 2016. There were a few exceptional periods where no blooms occurred, e.g., 1955 to summer of 1957; fall 1993, fall 2007 and fall 2010 (Fig 3). No samples were available from fall 1964-spring 1966, or from spring 1968—spring 1971. In addition, no samples were taken in fall 1982 and fall 1984, which is during the peak bloom season (Fig 2A) [77]. Notable blooms in 1971, 1978 and 1988 were sampled for only two months, and due to the lack of routine sampling at the time, we cannot determine if these were short-lived blooms or not.

The spatial extent and persistence of a bloom can vary greatly between years. Some areas are impacted continuously during the bloom, while others are impacted episodically. For example, the bloom that began in fall 1994 in the Sarasota area (around 27° N, Fig 1) persisted there until it declined in the fall of 1995 (Fig 2A). This occurred again in the fall of 2016, when the bloom started at Sarasota in September and remained there until April 2017. Similar persistence occurred in the same region in 2012. Other blooms impact much of the coast for a month or more; including the blooms that started in 1957, 1978, 1995, 2006, 2012, 2015, and 2018 (Fig 2B).

### 3.2. Bloom severity index

The annual BSI indicates that most severe blooms occurred from 1994 onwards (Fig 3). This likely reflects under-sampling prior to 1994. The 2002 bloom was the most severe bloom (BSI = 10; section 2.3) based on extent and duration of bloom presence, followed by 1995, 2001, and 2016. Another way to assess bloom severity considers the potential risk for respiratory impact (>50,000 cells L^-1^). Based on this criterion, 2001 would be the most severe bloom year. If the potential for severe respiratory and other impacts, such as fish kills are considered (>1,000,000 cells L^-1^) the most intense blooms occurred in 1994, 1995, 2012, and 2018 (Fig 3). In the under-sampled period, the 1973, 1978 and 1986 blooms were the most severe. In 1973, the >1,000,000 cells L^-1^ severity closely matched that seen in the most severe years after 1994.

The annual BSI provides a more direct measure of bloom severity for the entire bloom year, but it does not capture potential intense short-term impacts during the bloom revealed by the monthly BSI. For example, Feb 2016 was not only the peak month for BSI, but nearly all latitudinal bins in southwest Florida that month had bloom concentrations >100,000 cells L^-1^ (Fig 2b). The 2018 bloom, while not the most severe, had a particularly intense period, with the monthly BSI (>1,000,000 cells L^-1^) averaging 4.1 from June through November.

*Karenia brevis* cell counts are typically low at the end of the bloom year in June and July. However, there are instances where blooms from the previous fall do not abate by June and July and continue into the subsequent bloom year (which starts in August) (Fig 2B). These June and July blooms can be quite intense, exceeding 1,000,000 cells L^-1^. For example, this intensity occurred in June-July 2018 (BSI >1,000,000 cells L^-1^ of 2.3 and 4.6), June 1995 (3.1), July 1971 (3.1), June 1954 (1.5), July 2006 (1.5), June 1995 (0.8), July 1980 (0.8), July 1991 (0.8), June-July 2005 (0.8). When July blooms continued into August, such as in 1995, 2005 and 2018, they may have exacerbated the development of the typical fall blooms, resulting in the unusually intense fall blooms observed in each of these years.

The state of Florida has produced a table of presence of blooms each month from 1878 to the present for west Florida [78]. They identify months when either the inferred or measured concentration exceeded 100,000 cells L^-1^. The longest blooms reported by the state of Florida match this data set for the southwest Florida coast. Their longest blooms were 1994–1997 (30 months), 2002–2004 (21 months), 1953–1955 (18 months), 2004–2006 (17 months), 2017–2018 (15 months). The dataset presented here has nearly continuous blooms along the southwest Florida coast during all of these periods, indicating that blooms consistently impact this section of coast. The Florida table [78] does not identify a bloom in April and May 2002. However, the dataset presented here identified >100,000 cells in April, and a bloom present in May. This indicates that the longest bloom may actually be 31 months from Aug 2001 to Feb 2004.

### 3.3. Bloom seasonality

*Karenia brevis* blooms typically initiate in the fall of most years, and sometimes last through the winter, a result consistent with previous work [38, 79] (Fig 2A). In extreme cases the blooms persist into spring and summer. Blooms typically make the first landfall along the southwest Florida coast between 26.6º and 27.6º N in August or September (Fig 2A). This encompasses the region between Sanibel Island in the south to the entrance to Tampa Bay in the north (Fig 1). This region is impacted from September to January with blooms (>5,000 cells L^-1^) generally occurring in individual bins from 40% to 68% of the years (Fig 4A). North of 27.6° N (Pinellas County), blooms occur occasionally in August, but the frequency increases to October and November, then decreases slowly. South of 25.8° N, blooms are not observed until November (Fig 4A), and in two years (Fig 4A) they continued to spread further south to 25° N (Fig 2A). While blooms are clearly most common in the fall, there are bloom concentrations in the spring (May-July) of some years. A more detailed breakdown of the bloom severities by year are presented in S2 Table.

Cell concentrations >100,000 cells L^-1^, which cause moderate to severe respiratory irritation, follow a similar spatial and temporal frequency to that observed for >5,000 cells L^-1^, including the decline and southward migration of the bloom from December/January through March on average (Fig 4B). However, these concentrations occur no more than 44% of the years in any single bin (Fig 4B).

### 3.4. Respiratory irritation impact (RI) index

Respiratory irritation represents an immediate human impact of *K*. *brevis* blooms. With sufficient aerosolized toxins, virtually all people will respond with upper airway symptoms. Accordingly, the BCRS was used to develop monthly metrics of respiratory impact in Sarasota and Manatee counties. Observations were made at least 25 days per month for >85% the total months in the time series and observations for >20 days a month were available for 90% of the time series (Fig 5). Hence, the BCRS respiratory data provided a robust dataset for evaluating the magnitude of respiratory irritation impact (RI) caused by *K*. *brevis* blooms (Fig 6). Except for September-October 2015, the time series was continuous over the study period. The highest monthly RI (10) was in August 2018 followed by September 2018, September 2006, November 2018 and February 2017 (Fig 6). Bloom years in this time series when significant respiratory irritation occurred were 2006, 2016, 2017 and 2018, with 2018 the year with highest RI of the record (Fig 7). The most persistent respiratory irritation occurred in 2016–2017 and 2017–2018 (Fig 6). The irritation was relatively modest in 2012 and 2015, and quite limited in the remaining years.

The seasonality of RI (Fig 8) follows that of the BSI (between latitudes 27 and 27.6, Fig 4), with a peak in the fall, decreasing in winter and lowest frequency in the spring. However, while the RI has a peak frequency in September, the BSI shows a continuation of the peak frequency into October. The two plots show different information. Fig 4 (BSI seasonality) has the frequency of months that had a bloom present, while Fig 8 is the frequency of days (and beaches) in the month with respiratory irritation. There is a large difference between the two monthly frequency products. The maximum frequency of months having a bloom is 68% (Section 3.3), while the maximum frequency of days in each month with respiratory irritation was only 19%. The difference should be expected, as respiratory irritation is rare on days with offshore winds, and the blooms are patchy and do not occur at each beach [38].

### 3.5. Relationships among *Karenia* occurrence, respiratory irritation, and wind direction

**Fig 9:**
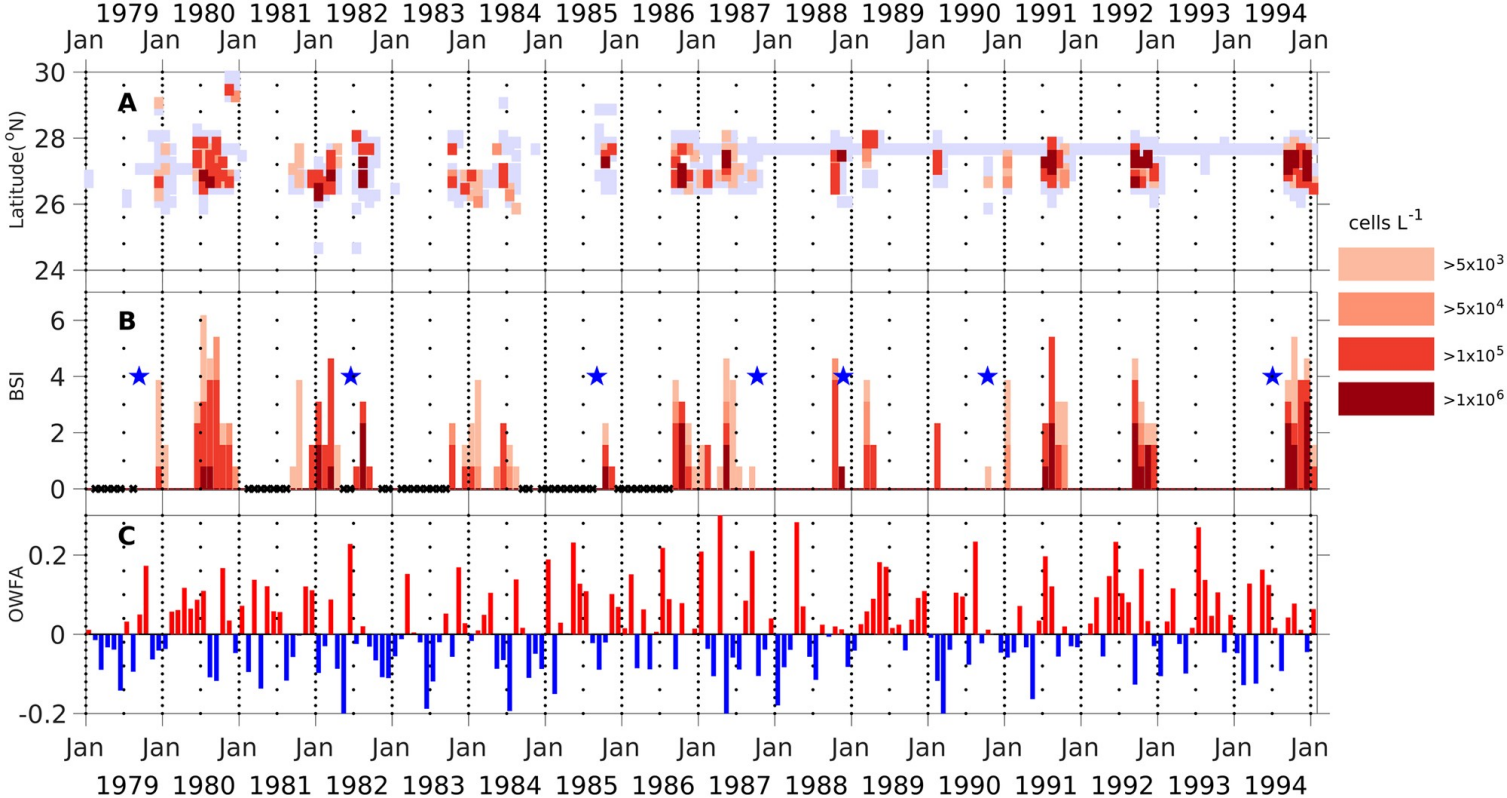
Comparison of binned monthly maximal bloom concentration and monthly severity with the monthly wind frequency anomalies for 1979–1984. (A) Hovmöller diagram for the maximal K. brevis cell counts in each 0.2° latitudinal bin for 25.4°-28.4° N for each month (Fig 2). (B) Normalized monthly bloom severity index (BSI) for cell counts in 1979–1994, accumulated from (A). (C) Proportional onshore wind frequency anomaly (OWFA) during each month from the data-assimilative NARR (North American Regional Analysis) product for the location of the VENF1 station (Fig 1). More Positive values are marked in red (more frequent onshore wind) and negative values (less frequent onshore wind) are marked in blue. Markers for the years indicate January 1. In (A), white indicates no samples, light blue gray indicates < 5,000 cells L-1. In (B), Xs on the X-axis indicate no samples were collected (prior to 1987), and the blue stars indicate times when tropical storms had impacts on southwest Florida coastal waters.

The periods when *K*. *brevis* blooms were poorly sampled between 1979 and 1994 generally correlated with periods where onshore winds were less frequent than usual, i.e. 1979, 1982–1983, 1984 and part of 1985 and to a lesser extent in 1981 (Fig 9). Correspondingly, in a number of the bloom events, sampling started during, or the month after, positive (onshore) wind frequency anomaly (OWFA) occurred and stopped after the onset of negative (offshore) OWFA.

In most years between 1979 and 1994, sampling started in conjunction with positive OWFA, and often did not occur during times when OWFA was negative. In fall 1979, there was a consistent positive OWFA before the bloom started (Fig 9). In 1980, increased sampling of the bloom started early (June 1980), following a persistent positive OWFA. In 1981, there were low to mild offshore wind anomalies when the blooms started (August 1981), then the OWFA turned highly positive as bloom sampling continued. This bloom continued through the winter 1981–1982 resulting in numerous manatee deaths [80]. The bloom was sampled and detected through April; Sampling ceased in May-June, 1982, following a period of negative OWFA in April-May. In September 1983 and May 1984, sampling, which started after occurrence of positive OWFA, detected a *Karenia* bloom. In fall 1984, there was no sampling during a period of persistent strongly negative OWFA. In 1985, despite a weak negative OWFA, sampling was initiated in September, but no bloom was detected. In October-November 1988, a bloom was present during a period when OWFA was negligible (near zero). Sampling decreased substantially in December, when OWFA turned negative, and stopped in January. Sampling restarted in March after a return to strongly positive OWFA. Sampling of the early bloom in July 1991 coincided with a change to strongly positive OWFA, which was preceded by a negative OWFA in the spring and weakly positive in June, neither of which were sampled. In 1993, limited sampling showed there was no bloom despite strong positive OWFA, suggesting that there was no bloom in 1993. In fall 1994, the start of sampling (and the observed bloom) occurred after a shift from negative to positive OWFA. In summary, before routine sampling started in 1995, increased onshore wind frequency (positive OWFA) appeared to be a catalyst for increased sampling in Southwest Florida, and that under-sampling of the bloom was consistent with periods of reduced onshore winds (negative OWFA).

### 3.6. Relationship between onshore winds and respiratory impact

**Fig 10:**
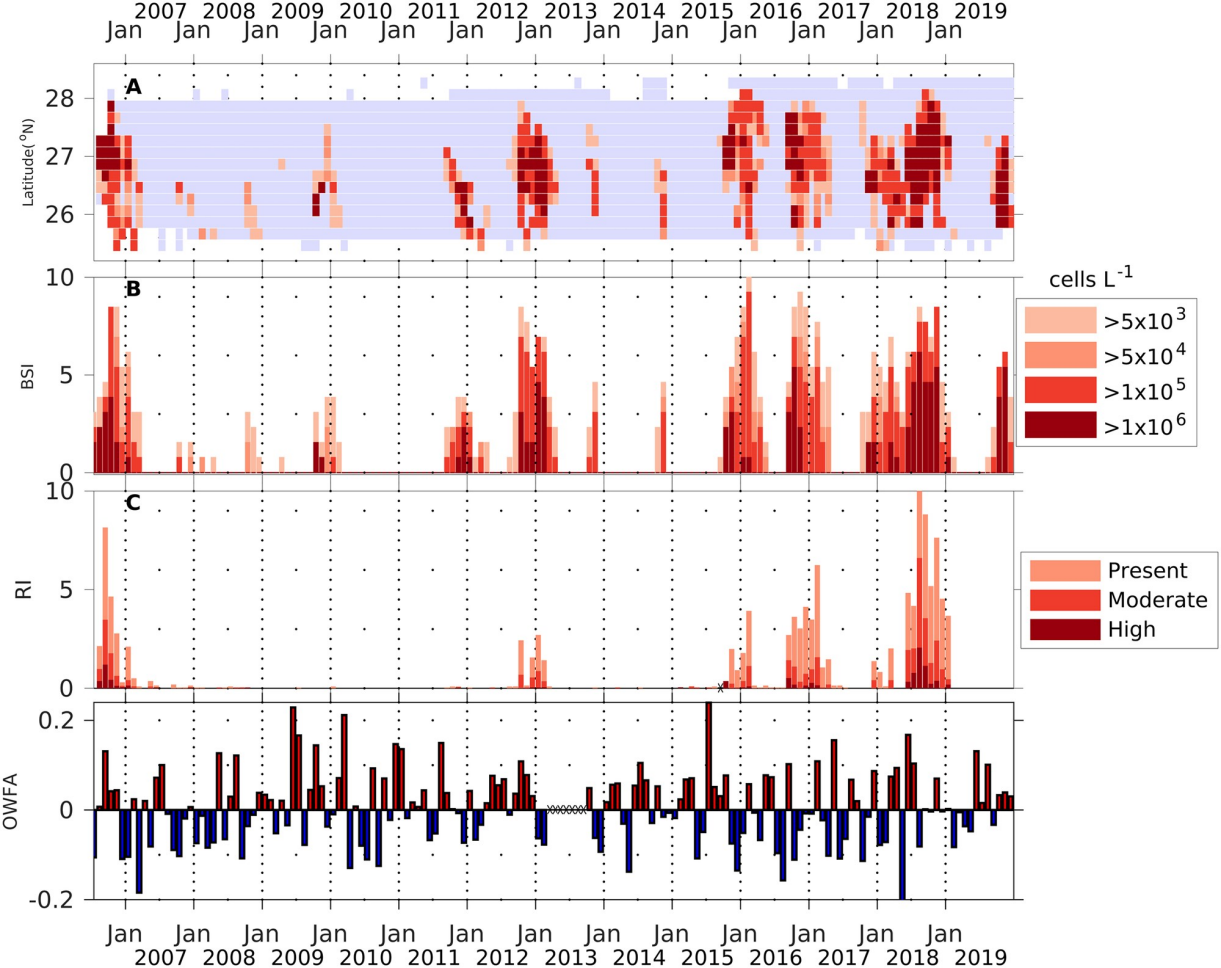
Comparison of binned monthly maximal bloom concentration, bloom severity index, respiratory index, and monthly wind frequency anomalies for 2006 to 2019. The figure covers the period of the respiratory irritation data set. (A) Hovmöller diagram for the monthly maximal *K*. *brevis* cell counts in each 0.2° latitudinal bin. (B) Monthly bloom severity index for the bloom year (August to July). (C) shows the monthly respiratory index for Manatee and Sarasota counties. (D) Monthly onshore wind frequency anomalies (OWFA) for Station VENF1 (Venice Pier, Florida), with positive values (more frequent onshore wind) marked in red and negative values (less frequent onshore wind) marked in blue. Markers for the years are for January 1. Descriptions are the same as Figs 6 and 9. Black Xs on X-axis during 2013 in the OWFA indicate no wind records were available.

Respiratory irritation impact (RI) generally corresponded with the size and intensity of the blooms represented by BSI (Fig 10). The largest blooms causing the most widespread respiratory irritation occurred in 2006, 2012, 2015, 2016, 2018 (Fig 10B). Years with mild blooms had little or no respiratory irritation (2007, 2008, 2009, 2010, 2013, 2014) (Fig 10B and 10C; S2 Table). Examining the monthly indices reveals most of the discrepancies that exist between the BSI and RI indices were related to wind anomalies (Fig 10D). For example, during a low to moderate BSI in September 2006, a high rate of respiratory irritation in September 2006 occurred (one of the highest observed in the time series), likely due to the strongly positive OWFA (more frequent onshore wind; Fig 10D). In contrast, a moderate bloom in fall 2011-spring 2012 only caused a limited respiratory impact and was associated with strongly negative OWFA (offshore wind anomaly).

**Fig 11:**
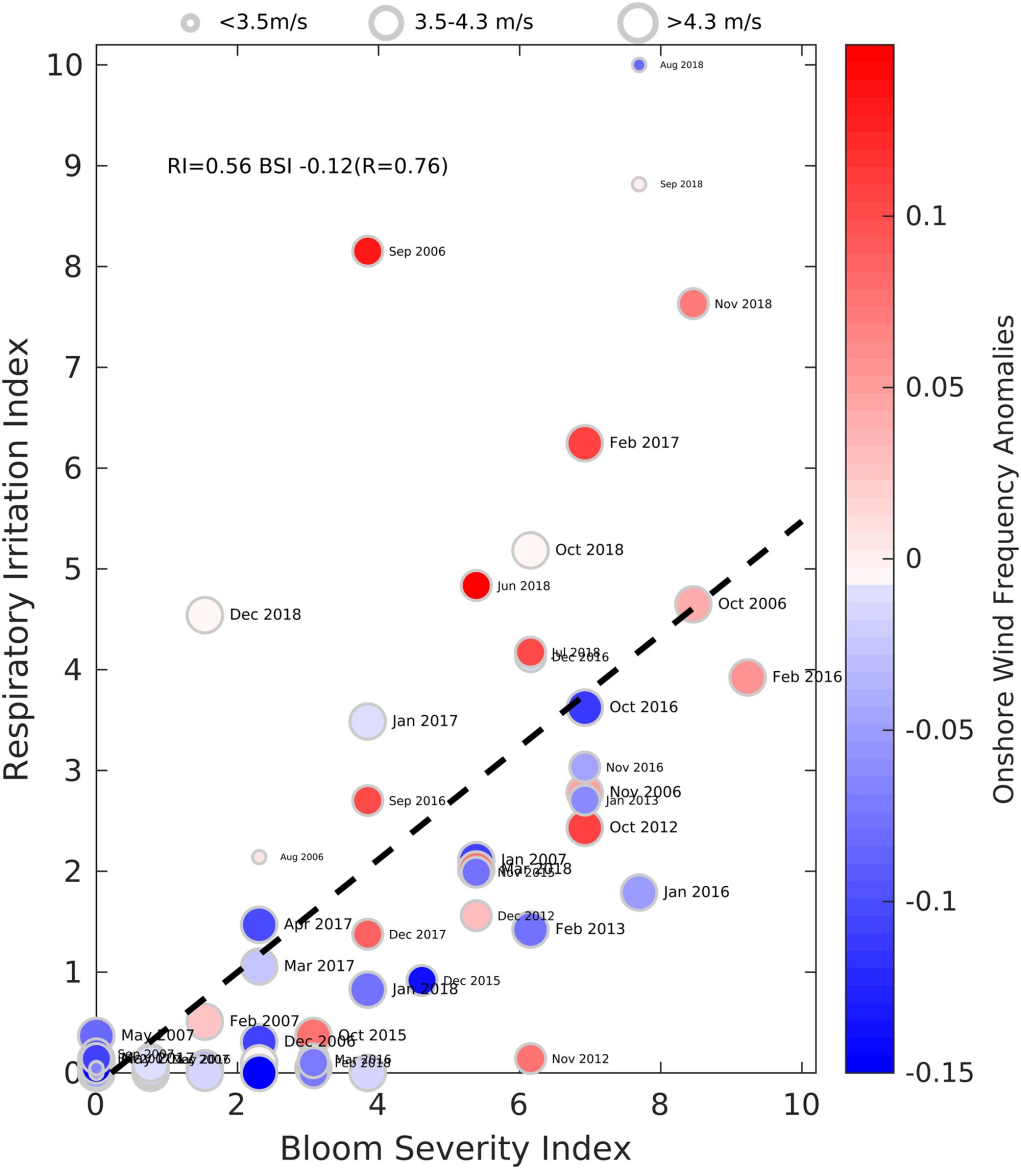
Relationship of monthly respiratory irritation impact (RI) index, monthly bloom severity index (BSI), and winds. The relationships are shown for cell counts > 50,00 cells L^-1^ for Manatee and Sarasota Counties, FL. Color-coding represents the onshore wind frequency anomalies (OWFA) for each month, positive (red) indicates onshore, and negative (blue) indicates offshore. Months when bloom were not observed were not shown. Size of the circles represent the wind speed for the month. April-June 2007 were not included because respiratory irritation during those months was caused by the Bugaboo Scrub wildfire. Black dashed line shows the linear regression fit between the monthly RI and BSI (equation shown).

The influence of wind on the discrepancies between RI and BSI are illustrated in Fig 11. Unsurprisingly, BSI and RI are correlated with a relationship of RI = 0.5 BSI. As expected, the deviations appeared largely related to wind anomalies. In cases with both BSI and RI indices >2, 13 of the 14 blooms above the regression line occurred during months with positive OWFAs. Below the line, 21 of the 32 blooms (66%) occurred during months with negative OWFAs. These results are consistent with onshore winds playing a critical role in augmenting the bloom impact along beaches. The one case when a bloom well above the line had a negative OWFA (predictive of mild RI) occurred in August 2018. However, this month also had one of the lowest average wind speeds in the record (Fig 11; the 2nd weakest was the following month, September) and it had one of the highest BSIs observed. This association of high BSI and weak winds suggests weak offshore winds were insufficient to ameliorate the aerosols and associated respiratory impacts produced by such high concentrations of *K*. *brevis* cells. A minimum offshore wind speed may be required to transport dense aerosols away from the beach during the strongest blooms. Most of the months when RI fell below the correlation line (milder impacts than expected for a given BSI) were associated with negative OWFAs (offshore winds). These results indicate high BSI blooms will have more severe respiratory impacts during positive OWFAs, and milder impacts during negative OWFAs.

## 4. Discussion

This study developed long-term, objectively-derived indices of *K*. *brevis* bloom severity (BSI) and respiratory irritation impact (RI). These indices were, respectively, applied to southwest Florida and to Sarasota and Manatee Counties; areas that consistently suffer from the adverse impacts of these blooms [25, 27, 78]. The BSI simultaneously characterizes *K*. *brevis* blooms in terms of spatial extent, duration, and cell concentrations. As such it has the potential to serve as a reference dataset for validating the accuracy of various models relating variations in bloom severity to environmental conditions. Feinstein [27] attempted to capture these components (extent, duration, and concentration), but was limited by the available data at the time. More recent models were validated only using cell concentration data, and not bloom extent or duration (see section 4.1). The long BSI time series will also support more robust analyses of models, as compared to using short validation time series [31]. The RI similarly provides a measure for evaluating the relative intensity of adverse bloom impacts for any region where such data sets are routinely collected (Figs 2 and 3).

Both indices are straightforward to update monthly or annually and can inform the public and resource managers of the relative severity of current or recent blooms. Similar indices were effectively used to provide severity information regarding the toxic cyanobacterial bloom in western Lake Erie [81] and for paralytic shellfish toxicity in the Gulf of Maine [64].

### 4.1. Relationship to other investigations

Prior to this study there were only limited *K*. *brevis* bloom ‘indices’ [31, 36, 49]. None of the indices addressed the combination of space, time, and concentration. Liu et al. [36] focused on presence of high concentration of the *K*. *brevis* after 1994, defining bloom presence by averaging for each week the top five maximal bloom concentration values found anywhere on the west Florida shelf. The index is useful for showing the bloom presence/absence in southwest Florida, but it did not capture extent or magnitude. For example, the bloom during fall 2007 was rather weak along the southwest coast of Florida (Fig 2), yet it was shown as ‘bloom present’ in Liu et al. [36], because of a bloom near the Florida Panhandle. With this type of presence/absence index, one cannot infer bloom impact on certain beaches and human health. Amin et al. [49] used satellite-derived chlorophyll-a fluorescence to estimate the area of blooms starting in 2002. While *K*. *brevis* is the dominant bloom-former in the fall [82], diatoms, which also fluoresce, can produce major blooms on the west Florida shelf [83, 84]. The method did identify major *K*. *brevis* blooms like those in 2005 and 2014, but it cannot distinguish *K*. *brevis* blooms from those caused by diatoms or other algae. Without additional validation against field measurements to confirm the presence of *K*. *brevis* blooms, the method cannot be used to reliably quantify bloom severity. For example, Amin et al. [49] presented that there was a large bloom in late 2010 that extended along the Florida panhandle; however, water samples showed no *K*. *brevis* cells present in that area [85].

### 4.2. Spatial and temporal patterns of bloom occurrence

This study provides more detailed information on bloom location and intensity along the southwest coast of Florida than previously available [20, 32] (Figs 2–4). Out of the 25 years starting in 1994, every year except 2010 had a bloom somewhere on this coast. Blooms most frequently occur between Sanibel Island and Tampa Bay from September to January (Figs 2A and 4). Consistent with previous studies, blooms were observed to initiate most frequently in August or September and terminate by late winter (Figs 2 and 4). The maximal monthly frequency of bloom occurrence was in November at Captiva Island, where blooms occurred 17 of the 25 years (68%). Only a few areas between Sanibel and Manasota Key (26.4° and 27° N) had months when blooms occurred in 50–60% of the years, while other areas experienced lower frequencies in all months. Concentrations greater than 100,000 cells L^-1^, which tend to cause respiratory impacts, have essentially the same seasonal and spatial distribution as bloom occurrence (> 5,000 cells L^-1^) (Fig 4). In many years, relatively strong blooms start with high concentrations (>1,000,000 cells L^-1^) over several bins of latitude, for example 1994, 2001, 2006, 2009, 2012, 2015, 2016, 2017. The blooms typically expand northward and southward in the months immediately after initiation, which is also evident in monthly frequency plots (Fig 4). However, that expansion is preferentially southward. This was particularly evident in 1954 and 2011–2012 as well as 1999, 2001, 2005, 2006 and 2017 (Fig 2A).

While blooms most often initiate from August into the fall months, there are exceptions. Continuation of previous year’s blooms through the summer and into the next fall occurred in 1954, 1987, 1995, 2002, 2003, 2005 and 2018 (Fig 2A). In most of these years, bloom intensity continued unabated throughout the summer, but in 2005 and 2018 the bloom re-intensified in June and July, respectively. There were also some years when the blooms started prior to August; these were June 1971, June 1980, May 1984, July 1991 and July 2006 (Fig 2A). All but one of these years occurred prior to 1994, when sampling was opportunistic. In all of these early years, except 1984, no samples were collected in the months before the bloom started (except the single monthly sampling location at New Pass after 1988). In 1984, the previous bloom continued until February, and the sampling was spatially inconsistent afterwards, so it cannot be determined if a new bloom started in May or if it was a continuation of the previous bloom. When these results are considered, with the seasonal frequency data, which shows a jump in bloom frequency in August, August continues to be an appropriate choice to define the start of the bloom season.

There are periods with consecutive years having either severe blooms or mild blooms. The recent years 2015–2018 all experienced intense blooms (Fig 3), as did 2001–2006. From 2007 to 2014, there was only one extreme bloom year, 2012. Similarly, 1996 to 2000 had relatively mild blooms. This type of variability may have existed prior to 1994, but is difficult to assess due to lack of data in some years. A topic for future research worth examining is whether a sequence of years with consecutive large (or small) blooms is due to a cyclical pattern of oceanographic or meteorological conditions or whether the observed patterns occurred by chance.

### 4.3. Correspondence between BSI and RI

A major question addressed in this study was the degree to which wind anomalies influence the respiratory impacts of *K*. *brevis* blooms (Figs 10 and 11). Years with severe blooms, such as 2006, 2012 and 2018, had noticeable respiratory irritation. Some years with blooms had almost no respiratory impacts (2008, 2009, 2011, 2013, 2014) (Fig 10). A portion of the scatter observed in Fig 11 can be attributed to spatial and temporal aliasing because we integrated over the month, and bloom samples and respiratory reporting are frequently not collected at the same location or at the same time. The BCRS observations are made daily at 8 fixed stations in Sarasota and Manatee counties and RIs were computed by accumulating all these observations. In contrast, bloom samples were collected weekly at some fixed stations (some of which correspond to BCRS, and some do not) and opportunistically at others, so the two data sets do not necessarily align. As a result, there may be aliasing, in that a bloom at a BCRS station might have been missed, or the most intense sample in the bin may have occurred for only a few days at another location within the 0.2 degree latitudinal bin. The difference between daily respiratory reports and binning of maximal monthly water samples for bloom detection may account for a bias toward higher BSI relative to the RI, with more months having onshore (reds) winds below the dashed line in Fig 11 than offshore winds (blues) above the line. Even with the potential for some spatial and temporal mismatch, the bulk data analysis clearly demonstrates the role of wind anomalies in moderating or enhancing the respiratory impact of *K*. *brevis* blooms.

### 4.4. Under-sampling of the fall blooms in the 1980s

Sampling was greatly reduced most years from 1962 through 1993 (Fig 2). As addressed in the introduction, much of the sampling during this period was initiated in response to adverse impacts commonly associated with blooms. From 1979 to 1993 (when NARR wind data sets are available), we have shown that under-sampling corresponds to periods of negative OFWA (reduced frequency of onshore winds) (Fig 9). From this result, it may be hypothesized that the sampling from 1962 to 1978 was also influenced by onshore wind frequency. However, the possibility that blooms did not occur in any of these years or that they were minimal cannot be ruled out. Despite this uncertainty during the earlier portions of the study, the near annual occurrence of blooms from 1994–2019 supports the argument that years without blooms are infrequent, with 1993 and 2010 being the only cases examples of bloom-free years on this coast (Section 3.5). The possibility of under-sampling caused by tropical storms was also examined and discarded. There was no correlation between hurricanes in the area (Stars in Fig 9B) and a lack of sampling. Ultimately, these data sets indicate many of the sampling deficiencies before 1994 may have been triggered due to wind ameliorating the respiratory impacts of *K*. *brevis* blooms. The time series of bloom intensity prior to 1994 thus likely reflects an under-estimate of the bloom occurrence and severity (Fig 3). This means that any claims that blooms have gotten worse over time are impossible to confirm given the sampling inconsistencies prior to 1994.

### 4.5. Offshore blooms

This study deliberately examined blooms at the coast because the consistent data collected in this region allowed construction of robust time series. Offshore blooms, in contrast, have historically only been sampled opportunistically, either during research programs, such as ECOHAB [86], or after reports of a large bloom (e.g., [32, 87]). Consequently, while the BSI accurately captured the scale of the blooms at the coast, as well as potential direct impact on people, it does not inform us about the potential offshore bloom impact. Blooms on the continental shelf can extend over much of the range occupied by both juvenile and adult fish, such as red grouper. These blooms cause significant mortalities and are of particular concern for fisheries management [88]. This means application of the BSI to evaluating offshore impacts on fisheries or aquaculture must be done judiciously.

Literature shows two offshore blooms that did not appear in the current time series, 2004 and 2014. The 2004 bloom formed offshore of southern Collier County (Naples, FL) and drifted southward in November and December. Though covering a large geographic region, the bloom never reached inshore waters [43]. The large 2014 bloom formed offshore of Pasco County (above 28º N) and briefly made landfall only along a portion of the northern Big Bend coastal region of Florida [85, 87] (Fig 2A). Blooms above 28.0º N are more sporadic and poorly sampled and are outside the geographic region covered in this application of the BSI (Fig 2A). Fully evaluating the mechanisms responsible for bloom formation in northern Florida, and any concomitant adverse impacts, would require instituting a more systematic sampling program.

### 4.6. Blooms do not impact the coast uniformly

The impact frequency of Florida red tides is usually presented in the context of how often blooms occur. It is typically stated that with only a few exceptions blooms occur annually [34, 74]. This assertion is supported by data from 1994 through 2018, where systematic sampling showed a bloom occurred in 24 of the 25 years (96%; Figs 2 and 3). In reality, blooms do not occur along the entire coast each year. Only 0.6 degrees (3 bins) of the west Florida coast (from Sanibel and Captiva Islands) had any months where >5,000 cells L^-1^ were present in >50% of the years sampled (Fig 4A). The highest frequency observed was 68% of years in the single bin centered on 26.7°N (north end of Captiva Island to north end of Boca Grande) in November. Cell counts >100,000 cells L^-1^ occurred no more than 44% of the time in any given month over the study region (Fig 4B).

When considering respiratory impacts, the contrast in frequency between annual bloom occurrence and the frequency of local impacts is even more striking. During the 12 years of respiratory monitoring, a bloom occurred somewhere in southwest Florida in 11 of those years (92%) (Fig 10). Yet over that time period, monitored beaches in Sarasota/Manatee counties (27.0º to 27.5º N) experienced no more than 18% of the days in any month on average with respiratory irritation (Fig 8). Lack of timely data on the location of blooms and respiratory impacts leads to “social amplification”, the public perception that the blooms and associated irritation occur nearly every year along all of the coast [89], which is clearly not the case. There is a large difference between perception and reality on how often blooms or bloom impacts occur.

### 4.7. Consideration for socio-economic studies

Hoagland et al. [7] conducted the most comprehensive analysis of an economic impact of *K*. *brevis* blooms to date. They used daily cell counts from two stations during blooms as a proxy for brevetoxin exposure in their analysis of changes in frequency of respiratory visits to emergency departments (ED). Though they recognized that onshore winds should be a factor in increasing exposure, they were unable to find a correlation between wind speed, direction, and ED visits. The results presented here indicate that more sophisticated analysis for wind impacts on brevetoxin exposure is required; that anomalies in onshore winds best describe respiratory impacts. Expanding the respiratory irritation sampling would provide more data for use in models designed to understand people’s response to blooms. Unfortunately, the respiratory irritation data set post-dates the Hoagland et al. study [7]. Analyses that combine cell counts, respiratory irritation (where available), and wind anomalies (e.g., Figs 10 and 11) should also improve future studies of economic modeling and analysis. Another application of this type of data is for siting of shellfish and finfish aquaculture operations that could suffer adverse impacts from these blooms. The seasonal analysis, combined with routine sampling along more of the coast, could be incorporated into approaches for identifying areas most favorable for aquaculture [90].

## 5. Conclusions

The recurring, toxic *Karenia brevis* blooms pose significant threats to the coast of Florida, yet to date there has not been a standard metric of bloom severity that is sufficiently flexible to accommodate spotty historical data and capture bloom dynamics at varied spatiotemporal scales. In this study, quantitative monthly and annual indices for *K*. *brevis* bloom severity (BSI) from 1953–2019 along the coast of Southwest Florida and associated respiratory irritation index (RI) in Manatee and Sarasota Counties from 2006–2019 are presented. The bloom severity and respiratory irritation indices consider not only the intensity (of cells or irritation), but also duration and extent along the coast relative to the most severe events on record, providing both spatial and temporal information not previously considered. These indices can directly benefit both environmental models of bloom dynamics and socio-economic assessments of *K*. *brevis* bloom impacts. The metrics and approach used here are not specific to SW Florida, and can be applied to any location that has routine sampling.

The BSI was based on the state cell count database from 1953–2019 and can provide an objective reference for evaluating models that attempt to determine bloom magnitude [27, 35, 36]. Because the BSI takes into account conditions within 5 km of the coast, our results may be useful in evaluating the overall contribution of land-based nutrient fluxes on the magnitude of *K*. *brevis* blooms.

Both BSI and RI developed in this study have shown the blooms and their respiratory impacts are not monolithic in time and space. While some locations might be affected for several consecutive months during severe blooms, the entire coast is not constantly impacted from initiation to termination (e.g., Figs 2, 4, 8 and 9). The bloom severity and respiratory irritation indices can be updated and extended in real-time to inform stakeholders and to aid other studies on *K*. *brevis*. These indices can help managers and decision makers evaluate the risks along the coast during a bloom but also within an interannual context, and help them plan ways to better respond to and mitigate bloom impacts.

This study shows higher-than-usual offshore winds suppress the respiratory impact of blooms (Fig 11). This association may have had a strong bearing on sampling prior to 1994, when sampling was reactive. Managers generally started monitoring when either respiratory impacts or dead fish were observed, both of which are dependent on onshore winds. Consequently, during periods of weak onshore winds, some blooms may have been undetected, or under-sampled. As a result, the BSIs before 1994 were likely underestimated in many years, meaning they cannot be viewed as accurately as those after 1994. These results emphasize that any assessment of increases in bloom severity along the Southwest coast of Florida that uses cellular abundance needs to start during or after 1994. The comparison of cell counts and respiratory impacts indicates that further expansion of routine respiratory monitoring beyond the counties examined here provides critical knowledge needed to better track, describe, and predict the impacts of these blooms.

## Supporting information

S1 Data A comma separated variable (CSV) file containing the concentration bin values by month that were used in this analysis. The values are cell counts per Liter. The headers are the central latitude of each 0.2° bin. NaN means there are no observations.
(CSV)

S2 Data A comma separated variable (CSV) file containing the monthly bloom severity index (BSI). Row is for each month, column is for BSI at each category (>5k cells/L, >50k cells/L, >100k cells/L, > 1million cells/L). NaN means there are no observations for that month.
(CSV)

S3 Data A comma separate variable (CSV) file containing the number of days each month at each station with respiratory irritation. Respiratory Irritation (Irritation Present, Moderate, High Irritation) conditions were reported for each beach. Row is for each month, column is each beach, first set is "present", second set is "moderate", third set is "high". NaN means there are no observations for that month.
(CSV)

S4 Data A comma separated variable (CSV) file containing the respiratory irritation index (RI) values by month. Row is for each month, column is for RI at each category (Irritation Present, Moderate, and High). NaN means there are no observations for that month.
(CSV)

S5 Data A comma separated variable (CSV) file containing the monthly wind frequency anomalies from 1979 to 1994. The columns are the NARR onshore wind frequency anomalies (OWFA) as proportion of winds having a positive or negative anomaly used in Fig 9. Row is for each month, column is the actual value for OWFA.
(CSV)

S6 Data A comma separated variable (CSV) file containing the monthly wind frequency anomalies and mean wind speeds from VENF1 for 2006–2019. Onshore Wind Frequency Anomalies- OWFA as proportions for the VENF1 meteorological station (Fig 1) for the periods in Fig 10, and the speed for wind (m s^-1^) as seen in Fig 11. Row is for each month, column is the actual value for OWFA. NaN means there are no VENF1 observations for that month.
(CSV)

S1 Fig The number of *Karenia brevis* cell count samples collected within 5 km offshore of the southwest Florida coast (Fig 1) for each year from 1953 to 2019. (TIF)

S2 Fig Hovmöller diagram for *K*. *brevis* cell counts from 1970–1980. (A) after filling to reduce missing data, and (B) data prior to the filling process. If a bin did not contain a sample, but the bins immediately adjacent in time and/or space did have samples, the empty bin was assigned (filled with) the average concentration of the adjacent bins. Color-coding represents maximum cell concentrations observed within each month in the grouped 0.2° latitudinal bins. Blooms were classified into 5 categories, <5,000 (light blue gray), >5,000, >50,000, >100,000, and >1,000,000 cells L^-1^. Lower panels (C and D) normalized bloom index for cell counts for the same period (as described in Section 2.2), with (C) as after neighbor infilling, and (D) as before the filling. Black Xs on the X-axis indicate months with no observations available. Markers for the years are for January 1.
(TIFF)

S3 Fig Hovmöller diagram for *K*. *brevis* cell counts from 1980–1990. A-D labels are the same as for S2 Fig.
(TIFF)

S4 Fig Calendar year bloom severity index (BSI) for each 25.4° to 28.4° N. Analogous to the bloom year index, the annual calendar bloom index was normalized by the maximum of the calendar year blooms and scaled to be 0–10 (0 as no bloom; 10 as largest bloom). Black Xs on the X-axis stand for bloom years with no available observations.
(TIFF)

S5 Fig Monthly (seasonal) climatology of *K*. *brevis* bloom frequency. (A) shows the frequency for > 50,000 cells L^-1^ and (B) shows >1,000,000 cells L^-1^. Color coding represents the percentage of years that the latitudinal bin had a concentration above the respective threshold (50,000 or 100,000 cells L^-1^). Numbers in each bin are the number of years (maximum 25). Gray indicates samples collected, but below the threshold. White with an X indicates no samples were ever collected in that latitudinal bin during that month of the year. X-axis runs from August to July to correspond to the bloom year.
(PNG)

S6 Fig Calendar-year respiratory irritation impact (RI) index for Sarasota County, Florida. The respiratory irritation index was defined by station days for each irritation condition (present, moderate+high, and high). This is an analog to the bloom-year respiratory irritation of Fig 7. It was then scaled by total number of station days within the year, normalized by the highest bloom so the index falls into the scale of 0–10 (zero as no respiratory irritation impact, and 10 as largest irritation impact).
(TIF)

S1 Table Data used to calculate the bloom severity index (BSI) and the respiratory irritation index (RI). The indices were calculated from the data sets in the table using the following formula: 10 × sum of latitudinal bins or beach-days / Maximal value of latitudinal bins or beach-days. Detectable concentration of cells refers to “low”, “moderate”, and “high” as described in the text. As an example, if a month had 3 bins in low, 2 in moderate, and 1 in high, the index (total normalized value) for the month would be 4.6, namely 10 × (3+2+1)/13, and the value for each category would be 2.3 (low), 1.5 (moderate), and 0.8 (high).
(DOCX)

S2 Table Summary of the years experiencing minor, moderate or extensive bloom events and respiratory irritation. (DOCX)
