# Supplementary figures and images for: Quantifying Karenia brevis bloom severity and respiratory irritation impact along the shoreline of Southwest Florida
Source: PLoS One. 2022 Jan 5;17(1):e0260755. doi: 10.1371/journal.pone.0260755 (PMC8730426; doi:10.1371/journal.pone.0260755)

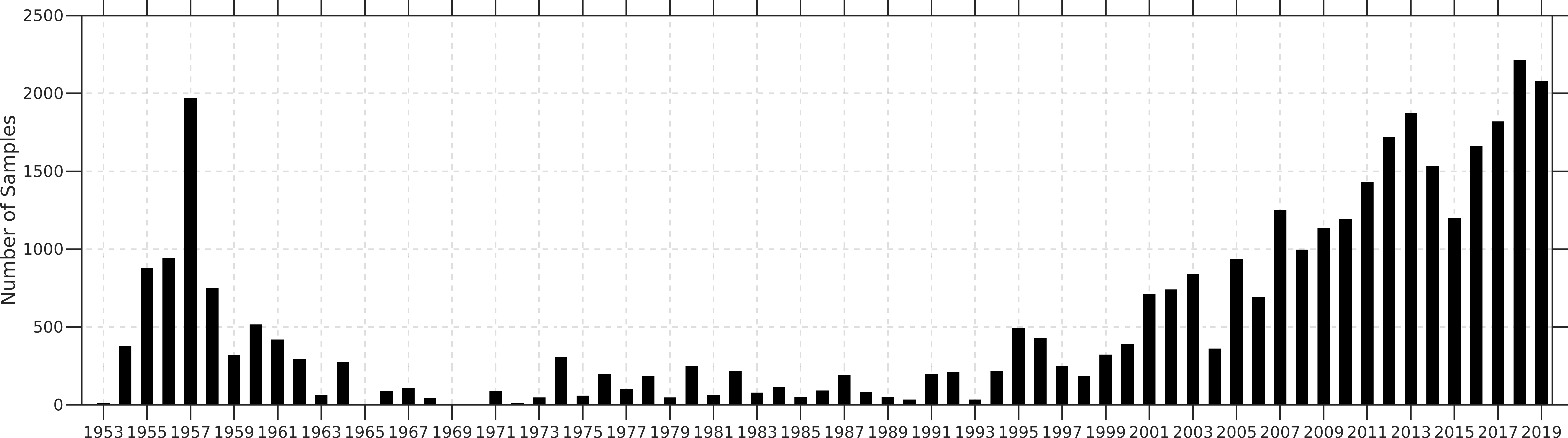

Supplement: S1 Fig — (TIF) [file pone.0260755.s007.tif]
