# Supplementary material for: Quantifying Karenia brevis bloom severity and respiratory irritation impact along the shoreline of Southwest Florida
Source: PLoS One. 2022 Jan 5;17(1):e0260755. doi: 10.1371/journal.pone.0260755 (PMC8730426; doi:10.1371/journal.pone.0260755)
Supplement: S1 Table — The indices were calculated from the data sets in the table using the following formula: 10 × sum of latitudinal bins or beach-days / Maximal value of latitudinal bins or beach-days. Detectable concentration of cells refers to “low”, “moderate”, and “high” as described in the text. As an example, if a month had 3 bins in low, 2 in moderate, and 1 in high, the index (total normalized value) for the month would be 4.6, namely 10 × (3+2+1)/13, and the value for each category would be 2.3 (low), 1.5 (moderate), and 0.8 (high). (DOCX) [file pone.0260755.s013.docx]

Quantifying *Karenia brevis* bloom severity and respiratory irritation impact along the shoreline of Southwest Florida

Supplementary Material

Richard Stumpf, Yizhen Li, Barbara Kirkpatrick, R. Wayne Litaker, Katherine A. Hubbard, Robert D. Currie, Katherine Kohler Harrison^,^ Michelle C. Tomlinson

**S1 Table. Data used to calculate the bloom severity index (BSI) and the respiratory irritation index (RI)**. The indices were calculated from the data sets in the table using the following formula: 10 × sum of latitudinal bins or beach-days / Maximal value of latitudinal bins or beach-days. Detectable concentration of cells refers to “low”, “moderate”, and “high” as described in the text. As an example, if a month had 3 bins in low, 2 in moderate, and 1 in high, the index (total normalized value) for the month would be 4.6, namely 10 × (3+2+1)/13, and the value for each category would be 2.3 (low), 1.5 (moderate), and 0.8 (high).

|  | Sum of latitudinal bins or beach-days | Maximal value of latitudinal bins or beach-days used for normalization (largest spatial extent of bloom or respiratory irritation reported during the study) | Total number of latitudinal bins or beach-days in the study area |
| --- | --- | --- | --- |
| Monthly BSI | Sum of bins in each month with a detectable concentration of cells | 13 bins (Feb 2016) | 18 bins |
| Annual BSI | Sum of bins over year with a detectable concentration of cells | 74 bins (2002) | 216 bins (18 bins x 12 months) |
| Monthly RI | Sum of all days in the month across all beach sites with measurable respiratory irritation | 218 beach-days (Aug 2018) | 248 beach-days (8 sites x 31 days) |
| Annual RI | Sum of all days in the year across all beach sites with measurable respiratory irritation | 856 beach-days (2018) | 2910 beach-days (8 sites x 365 days) |
