# Supplementary material for: Quantifying Karenia brevis bloom severity and respiratory irritation impact along the shoreline of Southwest Florida
Source: PLoS One. 2022 Jan 5;17(1):e0260755. doi: 10.1371/journal.pone.0260755 (PMC8730426; doi:10.1371/journal.pone.0260755)
Supplement: S2 Table — (DOCX) [file pone.0260755.s014.docx]

Quantifying *Karenia brevis* bloom severity and respiratory irritation impact along the shoreline of Southwest Florida

Supplementary Material

Richard Stumpf, Yizhen Li, Barbara Kirkpatrick, R. Wayne Litaker, Katherine A. Hubbard, Robert D. Currie, Katherine Kohler Harrison^,^ Michelle C. Tomlinson

**Table 1. Summary of the years experiencing minor, moderate or extensive bloom events and respiratory irritation.**

|  | Minor (<2) | Moderate (2 – 6) | Extensive (>6) |
| --- | --- | --- | --- |
| Bloom, 1994-2018 | 1996, 1997, 2000, 2008, 2009, 2010*, 2013, 2014 | 1998, 1999, 2003, 2004, 2005, 2007, 2011 | 1994, 1995, 1999, 2001, 2002, 2006, 2012, 2015, 2016, 2017, 2018 |
| Sarasota Blooms 2006-2018 | 2007, 2008, 2010, 2013, 2014 | 2009, 2011 | 2006, 2012, 2015, 2016, 2017, 2018 |
| Respiratory 2006-2018 **MISSING 2017** | 2007, 2008, 2009, 2010, 2011, 2013, 2014 | 2012, 2015 | 2006, 2016, 2018 |
| Bloom 1954-1993  Not sampled: 1965, 1968, 1969, 1970, 1984 | 1955, 1960-1966, 1974-1977, 1987-1991, 1993 | 1953, 1954, 1959, 1967, 1979, 1983, 1985, 1986, 1992 | 1957, 1973, 1978, 1980 |
| *2010 did not have a bloom | | | |
